# Integration of Genetic and Cytogenetic Maps and Identification of Sex Chromosome in Garden Asparagus (*Asparagus officinalis* L.)

**DOI:** 10.3389/fpls.2018.01068

**Published:** 2018-07-31

**Authors:** Roberto Moreno, Patricia Castro, Jan Vrána, Marie Kubaláková, Petr Cápal, Verónica García, Juan Gil, Teresa Millán, Jaroslav Doležel

**Affiliations:** ^1^Department of Genetics-ETSIAM, University of Córdoba, Córdoba, Spain; ^2^Institute of Experimental Botany, Centre of the Region Haná for Biotechnological and Agricultural Research, Olomouc, Czechia

**Keywords:** *Asparagus officinalis*, sex chromosome, FISH, flow-sorted chromosomes, SNPs, SSRs, genetic map

## Abstract

A genetic linkage map of dioecious garden asparagus (*Asparagus officinalis* L., 2*n* = 2*x* = 20) was constructed using F_1_ population, simple sequence repeat (SSR) and single nucleotide polymorphism (SNP) markers. In total, 1376 SNPs and 27 SSRs were used for genetic mapping. Two resulting parental maps contained 907 and 678 markers spanning 1947 and 1814 cM, for female and male parent, respectively, over ten linkage groups representing ten haploid chromosomes of the species. With the aim to anchor the ten genetic linkage groups to individual chromosomes and develop a tool to facilitate genome analysis and gene cloning, we have optimized a protocol for flow cytometric chromosome analysis and sorting in asparagus. The analysis of DAPI-stained suspensions of intact mitotic chromosomes by flow cytometry resulted in histograms of relative fluorescence intensity (flow karyotypes) comprising eight major peaks. The analysis of chromosome morphology and localization of 5S and 45S rDNA by FISH on flow-sorted chromosomes, revealed that four chromosomes (IV, V, VI, VIII) could be discriminated and sorted. Seventy-two SSR markers were used to characterize chromosome content of individual peaks on the flow karyotype. Out of them, 27 were included in the genetic linkage map and anchored genetic linkage groups to chromosomes. The sex determining locus was located on LG5, which was associated with peak V representing a chromosome with 5S rDNA locus. The results obtained in this study will support asparagus improvement by facilitating targeted marker development and gene isolation using flow-sorted chromosomes.

## Introduction

Garden asparagus (*Asparagus officinalis* L., 2*n* = 2*x* = 20) is the most economically important species of the *Asparagus* genus with a cultivation area similar to other vegetable crops such as garlic, carrot and eggplant (FAOSTAT, 2016). Edible asparagus are rich in minerals (potassium, phosphorus, calcium, and magnesium) and bioactive components, including flavonoids and saponins (Amaro-Lopez et al., 1995; Fuentes-Alventosa et al., 2007; Guillén et al., 2008; Vázquez-Castilla et al., 2013). It has been reported that saponins from edible spears induce *in vitro* typical features of apoptosis in human colon carcinoma cell (Jaramillo et al., 2016).

Garden asparagus is a dioecious perennial species and a single gene (*M/m*) is responsible for sex determination. Females are homozygous recessive (*mm*) whereas males are heterozygous (*Mm*) (Rick and Hanna, 1943) and the sex ratio in traditional clonal hybrids (*mm*: *Mm*) is thus 1:1. Sex is an important agronomical trait in this species because male plants are economically more profitable than female plants (Sneep, 1953; Benson, 1982; Ellison, 1986). Hence, the improvement of new cultivars focuses on development of ‘All–Male’ hybrids after crossing a female plant with a ‘supermale’ (*MM*). Supermales can be obtained after selfing andromonoecious plants, i.e., male plants that bear normal staminate and bisexual flowers. Andromonoecious plants occur spontaneously at low frequency. Supermales can also be obtained artificially from *in vitro* anther culture. However, the formation of androgenetic embryos is genotype dependent and the frequency of double haploid plants is generally low (Falavigna and Casali, 2002; Riccardi et al., 2011).

There are no morphological differences between male (*Mm*) and supermale (*MM*) plants and supermales are identified by sex ratio in segregating progeny derived from the cross between supermales and females. This process takes about 24 months in field conditions. To reduce the time needed to identify supermale plants, efforts have focused on development molecular markers linked to the sex locus. Accordingly, some dominant markers (Maestri et al., 1991; Restivo et al., 1995; Jiang and Sink, 1997; Reamon-Büttner et al., 1998; Jamsari et al., 2004; Nakayama et al., 2006; Gao et al., 2007; Kanno et al., 2014; Kim et al., 2014) and one co-dominant marker were reported (Jamsari et al., 2004). A co-dominant sex-linked marker would allow direct identification of supermales without test crosses. Unfortunately, the marker developed by Jamsari et al. (2004) is not functional in a number of genetic backgrounds (Nakayama et al., 2006).

To date, molecular marker development in asparagus focused on sex determination locus and only a few studies aimed at genetic mapping of agronomic traits (Anido and Cointry, 2008). Yet, there is an urgent need to map traits of agronomic importance, such as early yield, spear color, spear size, spear quality, tight heads, or disease resistances. Current genetic maps of asparagus are incomplete. They were originally constructed using isozyme markers and resulted in four detectable linkage groups (Maestri et al., 1991). Introduction of RFLP (restriction fragment length polymorphisms) markers and their mapping revealed seven linkage groups (Lewis and Sink, 1996). Later, a map integrating RFLP, RAPD (random amplified polymorphic DNA) and AFLP (amplified fragment length polymorphisms) markers, comprised ten linkage groups spanning 721.4 cM, with an average distance between markers being 2.6 cM (Spada et al., 1998). Recently, 65 SNPs (single nucleotide polymorphisms), 15 SSRs (simple sequence repeats) and the sex-linked marker were used to develop a genetic linkage map comprising 13 linkage groups (Mercati et al., 2013).

One approach to facilitate development of genetic maps and anchoring linkage groups to chromosomes is flow cytogenetics (Doležel et al., 2014; Vrána et al., 2016). DNA from flow-sorted plant chromosomes was shown suitable for a variety of applications, which included development of molecular markers from particular chromosomes in field bean (Požárková et al., 2002), wheat (Wenzl et al., 2010), and rye (Kofler et al., 2008) as well as anchoring DNA markers to chromosomes in barley (Muñoz-Amatriaín et al., 2011), garden pea (Neumann et al., 2002), and chickpea (Zatloukalová et al., 2011). Next generation sequencing DNA from flow sorted chromosomes enabled identification of a majority of genes in barley (Mayer et al., 2011), rye (Martis et al., 2013), and bread wheat (Mayer et al., 2014) and assign them to chromosomes. Later, this approach facilitated gene isolation in barley and wheat (Sánchez-Martín et al., 2016; Thind et al., 2017). Gene cloning and characterization in asparagus lagged behind other crops and to date, only a few genes were isolated. They mainly included some of the ones involved in the floral development (Park et al., 2003, 2004; Losa et al., 2004).

Sorting particular chromosomes by flow cytometry requires that they differ from other chromosomes in relative DNA content. The size of *A. officinalis* genome has been estimated to 1300 Mb/1C (Arumuganathan and Earle, 1991) and its chromosomes are relatively small (2.3–5.2 μm). Unfortunately, there is no general agreement on asparagus karyotype. Löptien (1976) classified asparagus chromosomes according to their length as long (L1–L5), medium (M1) and short (S1–S4). Löptien (1979) employed primary trisomics to assign sex locus to chromosome L5. Several groups mapped 5S and 45S rDNA genes to four chromosomes using fluorescence *in situ* hybridization (Reamon-Büttner et al., 1999; Melo and Guerra, 2001; Deng et al., 2012; Mousavizadeh et al., 2016). The reported differences between asparagus chromosomes size should make the species amenable to flow cytogenetics.

The aim of this work was to develop a new genetic map of *A. officinalis*, optimize a procedure for chromosome sorting using flow cytometry for this species and anchor genetic linkage groups to chromosomes by FISH and mapping SSR markers.

## Materials and Methods

### Plant Material

A mapping population of 175 individuals was obtained from a cross between genotypes PS010 and WN124 of *A. officinalis*. PS010 is a female plant of unknown origin and WN124 is a male plant from a wild accession from Asmundtorp (Skåne County, Sweden) which was kindly provided by the Nordic GenBank. The mapping population was phenotyped for sex. Seeds of *A. officinalis* cultivar ‘Mary Washington’ were employed for flow cytogenetic analyses.

### Genotyping by Sequencing (GBS)

DNA was extracted from both parents and each of the 175 individuals of the mapping population using DNeasy Plant kit (Qiagen, Hilden, Germany) and the samples were genotyped by tGBS variant of genotyping-by-sequencing (Data2Bio, Ames, United States). As there was no reference genome available for aligning the raw reads for SNP discovery at the time of this work, a reference-free analysis was conducted. Prior to alignment, the nucleotides of each raw read were scanned for low quality bases. Bases with Phred quality scores <15 (out of 40) (Ewing and Green, 1998; Ewing et al., 1998) were trimmed by a custom pipeline. Then, consensus sequences that could be used *in lieu* of a reference genome for alignment and SNP calling were generated. Trimmed sequence reads from all samples were combined and normalized to a maximum of 50× coverage, using diginorm (Brown et al., 2012). Sequence read errors were then corrected using Fiona (Schulz et al., 2014). The coverage-normalized and error-corrected reads were then condensed using cd-hit-454 (Fu et al., 2012) with ≥95% identity to form consensus clusters. Clusters with <10 component reads and <50 bp in length were discarded in a final clean-up. Subsequently, the trimmed reads from each sample were aligned to the consensus reference sequence using GSNAP (Wu and Nacu, 2010) and confidently mapped reads were filtered if mapped uniquely (≤2 mismatches every 36 bp and less than five bases for every 75 bp as tails) and used for subsequent analyses.

Single nucleotide polymorphism calling was conducted using the uniquely aligned reads that had ≥5 reads in at least 50% of the samples. A SNP site was called homozygous in a given sample if at least five reads supported the major common allele at that site and at least 90% of all aligned reads covering that site shared the same nucleotide. A SNP was called heterozygous in a given sample if at least one read supported each of at least two different alleles and each of the two allele types separately comprised more than 20% of the reads aligning to that site. Also, the sum of the reads supporting those two alleles at least had to be equal to five and comprised at least 90% of all reads covering the site. In a cross-pollinated mapping population, a given SNP could be heterozygous in one parent and homozygous in the other parent or heterozygous in both parents. Hence, the SNPs were categorized into three subgroups (a) pseudo-backcross PS010 SNPs (heterozygous only in WN124), (b) pseudo-backcross WN124 SNPs (heterozygous only in PS010) and (c) pseudo-F_2_ SNPs (heterozygous in both parents). SNP markers segregating either 1:1 or 1:2:1 were used for map construction. SNP markers derived from tGBS were coded with a number indicating the scaffold followed by an underscore and a number indicating the physical SNP position on the scaffold (i.e., 336532_89).

### Genotyping Using Microsatellite (SSR) Markers

A set of 97 SSR markers (Caruso et al., 2008; Mercati et al., 2013; Li et al., 2016) was first tested for polymorphism between the parents of the mapping population and then entire population was genotyped with those that were polymorphic. SSR markers used in this study are listed in Supplementary Table S1. PCR reactions were performed as described by Caruso et al. (2008); Mercati et al. (2013) and Li et al. (2016). Forward primers were tailed with the M13 sequence (TGTAAAACGACGGCCAGT) at the 5′ end (Schuelke, 2000) and PCR products were sized by capillary electrophoresis on ABI3130 Genetic Analyzer (Applied Biosystems/Hitachi, Madrid, Spain). Data generated were collected and analyzed using Genescan and Genotyper (Life Technologies, Foster City, CA, United States). Chi-squared test was performed to test for different segregation ratios on all scored markers. Markers that did not segregate in the ratio expected for F_1_ cross from outbreeding parents (either 1:1, 1:2:1 or 1:1:1:1) were excluded from linkage analysis (Supplementary Table S1).

### Genetic Linkage Map Construction

Single nucleotide polymorphism, SSR and data on plant sex were combined and formatted for linkage mapping using the standard codes for a cross-pollinated population of JoinMap v4.0 (van Ooijen, 2006). Markers which were heterozygous in only one of the parents were scored either <lm × ll> or <nn × np> depending on the parent, and heterozygous markers in both parents with two alleles were scored as <hk × hk>. Markers that were heterozygous in both parents with three and four alleles were scored as <ef × eg> and <ab × cd>, respectively. Markers with identical genotypes in the progeny were grouped into mapping bins of identical genotypes and a single genotype for each bean was used for map construction, and markers with more than 10% missing data were excluded from linkage analysis. Loci were then organized into parental sets, and maternal (markers segregating as <lm × ll>, <hk × hk>, <ef × eg> and <ab × cd>) and paternal maps (markers segregating as <nn × np>, <hk × hk>, <ef × eg> and <ab × cd>) were analyzed separately. Data were grouped into linkage groups (LG) using a minimum LOD score of six and maps were constructed using the maximum likelihood mapping algorithm. All other settings were default. Linkage maps were drawn using MapChart 2.2 software (Voorrips, 2002).

### Preparation of Suspensions of Intact Mitotic Chromosomes

The procedure of Doležel et al. (1992) was used with modifications. Briefly, young seedlings with 0.8 – 1.2 cm-long primary roots were transferred to Hoagland’s nutrient solution (Hoagland and Arnon, 1950) supplemented with 3 mM hydroxyurea (HU) and incubated for 18 h. In order to accumulate dividing cells in metaphase, the seedlings were first transferred to HU-free Hoagland solution and incubated for 5 h and then transferred to the solution supplemented with 5.0 μM oryzalin (Eli Lilly and Co., Indianapolis, IN, United States). All treatments were made in the dark at 25° ± 0.5°C and all solutions were aerated. The synchronized root tips were immediately fixed in 2% (v/v) formaldehyde at 5°C for 20 min and then washed three times for 5 min in Tris buffer. In order to isolate mitotic metaphase chromosomes, meristem tips were dissected and homogenized using a Polytron PT 1300 homogenizer (Kinematica AG, Litau, Switzerland) at 13,000 rpm for 18 s in LB01 buffer (Doležel et al., 1989). The crude homogenate was filtered through a 50-μm nylon filter to remove large cellular debris.

### Flow Cytometric Chromosome Analysis and Sorting

Suspensions of isolated chromosomes were stained with DAPI at final concentration of 2 μg/ml and analyzed at rates of 1000– 2000/s on FACSAria SORP flow cytometer and sorter (Becton Dickinson, San José, CA, United States) equipped with solid-state UV laser. Relative fluorescence intensities of DAPI-stained chromosomes were acquired on histograms of fluorescence pulse area (FL1-A), which were gated from dot-plot of fluorescence pulse height (FL1-H) vs. forward scatter (FSC). Chromosomes were sorted from dot-plots of FL1-A vs. fluorescence pulse width (FL1-W) into 40 μl of sterile deionized water in a 0.5 ml PCR tube. Three independent samples of 100,000 chromosomes were sorted from each peak. DNA of sorted chromosomes was amplified using illustra GenomiPhi V2 DNA Amplification Kit (GE Healthcare, Piscataway, NJ, United States) following Šimková et al. (2008). The independent amplification products from each peak were combined to reduce a potential amplification bias.

### Identification of Flow-Sorted Chromosomes by Fluorescence *in Situ* Hybridization (FISH)

The chromosome content of each peak on flow karyotype was determined after analyzing 1000 chromosomes flow-sorted onto a microscopic slide. The chromosomes were identified by fluorescence *in situ* hybridization (FISH) as described by Kubaláková et al. (2003) using probes for 5S and 45S rDNA. Biotin-labeled probe for 5S rDNA was prepared using PCR with a pair of specific primers (RICRGAC1, RICRGAC2) with rice genomic DNA as a template (Fukui et al., 1994). A pTa71 probe labeled with digoxigenin was used to map 45S rDNA genes (Gerlach and Bedbrook, 1979). The sites of digoxigenin-labeled probe hybridization were detected by anti-digoxigenin-FITC (Boehringer Mannheim, Mannheim, Germany) and the signal was amplified with anti-sheep-FITC (Vector Laboratories, Burlingame, CA, United States). Biotin-labeled probe was detected by Cy3-labeled streptavidin and amplified biotinylated anti-streptavidin (Vector Laboratories). The preparations were counterstained with 0.2 mg/ml propidium iodide (PI) and mounted in Vectashield antifade solution (Vector Laboratories).

### Assignment of SSR Markers to Flow-Sorted Chromosome Fractions

DNA amplified from flow-sorted chromosomes was used as template for PCR with primers for 76 SSR markers (Caruso et al., 2008; Mercati et al., 2013; Li et al., 2016). PCR was carried out in 10 μL reaction mix containing 10 ng chromosomal DNA, 5x Taq polymerase buffer, 0.2 mmol/l dNTPs, 2 mmol/l MgCl_2_, 0.25 μM of each of the forward and reverse primers and 0.25 units of Taq polymerase (Promega, Madison, United States). PCR was done using a Perkin Elmer Cetus DNA Thermal Cycler (9600) programmed as follows: an initial 2 min denaturation at 94°C; 30 cycles of denaturation at 94°C for 1 min, annealing at 55°C for 1 min, and extension at 72°C for 3 min; followed by a 10 min final extension at 72°C. PCR products were analyzed by electrophoresis on 2% agarose gels and visualized by ethidium bromide staining. The assignment of each SSR locus to individual chromosome peak was done according to the presence or absence of the PCR product.

## Results

Sequencing the tGBS library constructed from the mapping population PS010 × WN124 generated 624.4 million reads. Their number ranged from 149.017 to 8.3 million and averaged 2.6 million per sample. After trimming low quality bases, around 90% of sequence reads were used for SNP discovery. A total of 9109 segregating SNPs were identified in the mapping population after the analysis and filtering of tGBS data. Of these, 3766 were heterozygous only in PS010 parent, 3347 were heterozygous only in WN124 parent, and 1996 were heterozygous in both parents.

Of the 97 SSR markers checked for polymorphism between the parents of the mapping population, 57 were found homozygous in both parents, or gave ambiguous results, 16 were heterozygous in PS010 parent, 13 were heterozygous in WN124 parent, and 11 were heterozygous in both parents (Supplementary Table S1).

The segregating SNPs were combined with the SSR markers in one data set. After the exclusion of markers with more than 10% of missing data, those with significant segregation distortion, and those with identical genotype for all samples, a total of 1403 markers (1376 SNPs and 27 SSRs) were selected for linkage analysis and map construction. Of the 1403 markers used for map construction, 1219 segregated 1:1, 178 segregated 1:2:1 and six segregated 1:1:1:1. The population was also phenotyped for plant sex and the results followed the expected 1:1 segregation ratio (82 female, 84 male, χ^2^ = 0.02, *P* = 0.87). A genetic linkage map was constructed for each parent and the results are summarized in **Table 1**. The PS010 (female) map consisted of 907 markers distributed across 10 linkage groups. The WN124 (male) map comprised the phenotypic marker for sex and 678 markers distributed in 10 linkage groups. The phenotypic marker for sex was located on LG5 of the male parent WN124. Associations between the PS010 and the WN124 maps were identified using markers heterozygous in both parents (hk × hk, ef × eg, and ab × cd). These markers mapped to linkage groups in both parents. All linkage groups from PS010 were aligned with WN124 by this approach.

**Table 1:** Summary of PS010 × WN124 genetic linkage maps.

Linkage group	Female parent PS010			Male parent WN124		
	No. SNP	No. SSR	Total length (cM)	No. SNP	No. SSR	Total length (cM)
1	42	0	106	56	0	159
2	53	2	104	59	0	122
3	98	1	160	72	0	108
4	86	1	225	46	1	144
5	110	4	238	55	2	201
6	64	2	199	109	2	236
7	101	3	250	95	6	345
8	151	2	273	36	0	173
9	54	1	146	43	0	111
10	130	2	246	92	4	215

	889	18	1947	663	15	1814

**FIGURE 1:**
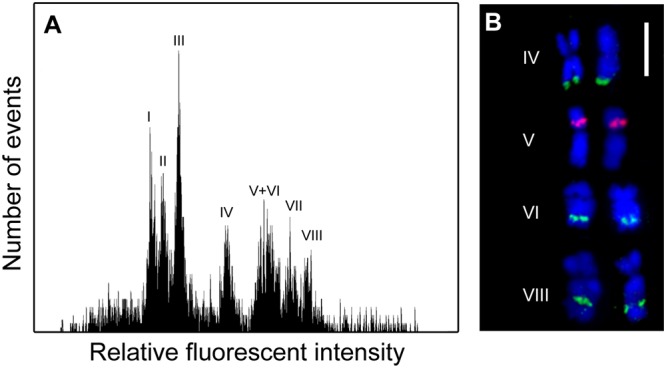
Flow cytometric chromosome analysis and sorting in garden asparagus (*Asparagus officinalis*, 2*n* = 2*x* = 20). **(A)** Histogram of relative fluorescence intensity (flow karyotype) obtained after the analysis of DAPI-stained chromosomes. Eight major peaks (I–VIII) could be resolved. **(B)** Examples of chromosomes sorted from peaks IV, V, VI, and VIII. The chromosomes were identified after fluorescence *in situ* hybridization/FISH with probe for 45S rDNA (green) and 5S rDNA (red) loci. The chromosomes were counterstained with DAPI (blue). Scale bar: 5 μm.

Flow cytometric analysis of DAPI stained chromosomes resulted in flow karyotypes with eight major peaks (**Figure 1A**). In order to characterize chromosome content of individual peaks, chromosomes were sorted onto microscope slides and the presence of 45S and 5S rDNA sequences were verified using FISH (**Figure 1B**). Relative chromosome length corresponded to the position of peaks on flow karyotype with the smallest chromosome sorted from peak I and the largest chromosome sorted from peak VIII. FISH analysis revealed that chromosomes sorted from peaks IV, VI and VIII carried 45S rDNA signals, albeit with different location: terminal in peak IV, and subterminal in peaks VI and VIII. Peak V contained chromosomes with 5S rDNA sequences.

**FIGURE 2:**
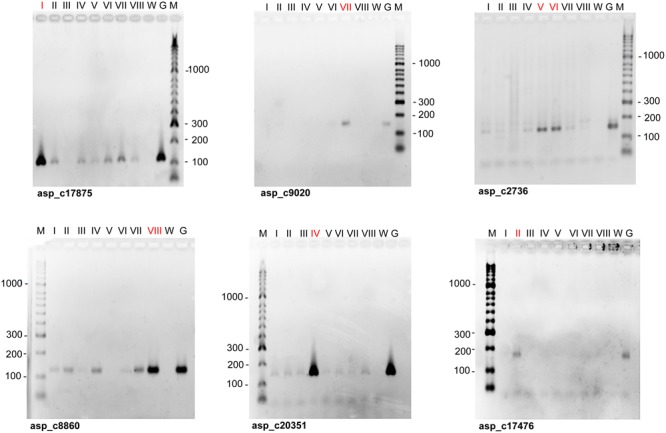
Examples of the assignment of SSR markers to chromosomes of garden asparagus. Agarose gel electrophoresis was done with PCR products obtained with primers for SSR markers (asp_c) and DNA of chromosomes flow sorted from eight major peaks on the flow karyotype (**Figure 1A**) as templates (lanes I – VIII). The list of markers is given in Supplementary Table S1. The numbers of positive peaks are printed in red. Genomic DNA (lanes G) and distilled water (lanes W) were used as positive and negative controls, respectively. Lanes M: DNA ladder HyperLadder^TM^ 50 bp (Bioline, London, United Kingdom).

Seventy-two out of the 76 SSR markers were useful to characterize individual peaks on the flow karyotype. The remaining four markers were discarded due to ambiguous amplification. Sixty-one out of the 72 markers were assigned to a single chromosome peak, while the remaining eleven markers produced amplification products in peaks V and VI, and therefore were assigned to both peaks. Examples of mapping SSR markers to peaks on the flow karyotype using PCR with primers specific for the markers and DNA of chromosomes flow sorted from peaks on the flow karyotype are shown in **Figure 2**. The number of SSR markers assigned to individual peaks ranged from three (Peaks I and II) to eleven (Peaks V and VI) (**Table 2**).

**Table 2:** Assignment of 72 SSR markers to individual peaks on flow karyotype of *Asparagus officinalis*.

Peak I	Peak II	Peak III	Peak IV	Peak V	Peaks V and VI	Peak VI	Peak VII	Peak VIII
asp_c17875	asp_c17476(LG2)	asp_c6290	asp_c1367	AG3(LG5)	asp_c13301(LG10)	TC7(LG6)	asp_c4789(LG7)	asp_c9810(LG8)
asp_c5587	asp_c17769(LG2)	asp_c12534(LG9)	asp_c20351	asp_c2370	asp_c2736(LG10)	asp_c14231(LG6)	AG8(LG7)	asp_c11969(LG8)
asp_c6470	TC6	asp_c2771	asp_c21312	AG7(LG5)	asp_c1390(LG10)	AG10(LG6)	TC9(LG7)	asp_c20893
		asp_c6790	ssr15(LG4)	asp_c1401(LG5)	AG2(LG10)	asp_c1319	asp_c22357(LG7)	asp_c957
		asp_c2122		asp_c9454	asp_c1505	asp_c753	asp_c923(LG7)	asp_c22306
		TC4		asp_c17381	asp_c15627	asp_c12796	asp_c11938	AG5
		asp_c3803		asp_c45	AG11	asp_c2065	asp_c4593	asp_c8860
		asp_c16828		asp_c11979(LG5)	asp_c3091	asp_c8724	TC2	AAT1
		ssr69(LG3)		TC5	asp_c1779	AG12	asp_c9020	AGA1
				asp_c8280	asp_c2848	TC8	asp_c6215(LG7)	AG6
				TC1(LG5)	TC3(LG10)	asp_c10809(LG6)		

*LG to which the markers belong according to the PS010 × WN124 linkage map are indicated in brackets. Markers without LG assigned were not polymorphic in the population. Markers names prefaced with asp were generated by Mercati et al. (2013), those prefaced with ssr were generated by Li et al. (2016) and the remaining markers were developed by Caruso et al. (2008).*

The genetic map of the PS010 × WN124 population has ten linkage groups, corresponding to the haploid chromosome number of asparagus (Supplementary Figure S1). The linkage groups were assigned to chromosome based on the markers which were identified in each peak on the flow karyotype (**Table 2**). SSR loci were distributed throughout all linkage groups but one of the PS010 × WN124 maps, and thus LG1 was assigned to peak I by default. Two different linkage groups (LG9 and LG3; identified by markers asp_c12534 and SSR69, respectively) were assigned to peak III, indicating that the peak contains two different chromosomes. LG5 and LG6 have markers assigned to peaks V and VI, respectively. LG10 comprised markers that were assigned to the neighboring peaks V and VI. The linkage map results indicated that markers assigned to peaks V and VI were located in three different LGs. Thus, the region of peaks V and VI most probably represents three different chromosomes. The four remaining linkage groups (LG2, LG4, LG7, and LG8) were unambiguously assigned to peaks II, IV, VII, and VIII, respectively. These peaks were clearly discriminated on the flow karyotype and represent only one chromosome each, confirming the results obtained by linkage analysis.

## Discussion

We have constructed a genetic linkage map of garden asparagus using SSR and SNP markers. To date, only 97 SSRs were developed in this species (Caruso et al., 2008; Mercati et al., 2013; Li et al., 2016). Some of them were employed in phylogenetic studies (Castro et al., 2013), characterization of genetic diversity, cultivar identification (Caruso et al., 2008, Castro et al., 2014; Li et al., 2016) and in genetic mapping (Mercati et al., 2013). However, the construction of high-density genetic maps based on SSRs only may not be realistic due to the enormous amount of work needed and prohibitive cost. On the other hand, the decreasing cost of the next generation sequencing together with the advances in high-throughput detection of SNP loci using GBS and the improvement of bioinformatics tools, made SNP markers increasingly useful for genetic map construction. We have used tGBS (Schnable et al., 2013) in this work to identify and genetically map a significant number of SNPs in asparagus.

With the aim to anchor genetic linkage groups to individual chromosomes and develop a tool to facilitate genome analysis and gene cloning, we have optimized a protocol for asparagus chromosome analysis and sorting using flow cytometry. We have obtained histograms of relative fluorescence intensity (flow karyotypes) comprising eight major peaks. Considering the basic chromosome number of *A. officinalis* (*x* = 10), a majority of peaks should represent one of its ten chromosomes. The eight chromosome peaks could be classified into three groups: (i) peaks I–III, (ii) peak IV and (iii) peaks V–VIII, which seem to correspond to the three chromosome groups of the asparagus karyotype formula: four small size (S1–S4), one medium size (M1) and five large (L1–L5) (Löptien, 1979). This observation suggests that relative fluorescence intensity of DAPI-stained chromosomes is proportional to their length. If so, four out of the ten asparagus chromosomes could be sorted from peaks I–III. Considering the area of peak III relative to peaks I and II, peak III should represent two different chromosomes. Thus, peaks I and II as well as peak IV should represent single chromosomes. The remaining five chromosomes should be represented by peaks V–VIII.

A majority of SSR markers employed in this work were useful to characterize flow-sorted chromosome fractions. Out of them, 11 SSR markers were assigned to chromosomes sorted from peaks V and VI. This observation could be explained by cross-contamination between peaks V and VI due to their overlap. However, it is highly probable that there is additional peak representing another chromosome between the peaks V and VI, which has intermediate relative fluorescence intensity and cannot be resolved. The fact that SSR markers from a single linkage group (LG10) mapped to chromosomes sorted from peaks V and VI seems to support this assumption and suggests that the region of the flow karyotype delineated with peaks V – VI contains three different chromosomes. Based on these observations we conclude that single chromosomes can be sorted from five peaks (I, II, IV, VII, and VIII), two chromosomes from peak III and three chromosomes from the region of peaks V and VI.

Currently it is thus possible to sort individually 5 out of the 10 asparagus chromosomes. Further improvement of the protocol, e.g., by employing FISHIS (Giorgi et al., 2013) with appropriate microsatellite probes may increase the number of chromosomes that can be discriminated and sorted. An alternative approach is to sort and sequence single chromosomes, which avoids the need for chromosome discrimination and provides DNA sequences from the chromosome of interest free of contamination by other chromosomes (Cápal et al., 2015). However, this strategy is not suitable for preparation of high molecular weight DNA, which is needed for long-read DNA sequencing technologies and optical mapping.

In previous studies, 45S rDNA genes were localized on three different chromosomes in a haploid set of *A. officinalis* whereas in wild species of genus *Asparagus*, including *A. persicus* (2x), *A. verticillatus* (2x), *A. prostratus* (4x), and *A. maritimus* (6x) two loci per basic chromosome set (x) were identified (Reamon-Büttner et al., 1999; Melo and Guerra, 2001; Moreno et al., 2005; Deng et al., 2012; Mousavizadeh et al., 2016). We observed 45S rDNA loci on three chromosomes per haploid set, confirming the previous results. These chromosomes were sorted from peaks IV, VI, and VIII.

The localization of 5S rDNA probe on chromosomes sorted only from peak V agrees with previous studies in which one locus per haploid chromosome set was observed (Reamon-Büttner et al., 1999; Mousavizadeh et al., 2016). In contrast, Deng et al. (2012) identified four and three loci for 5S rDNA and 45S rDNA, respectively, in *A. officinalis*. However, the authors used the same fluorescent label to detect 45S and 5S probes and this could lead to erroneous conclusions. Mousavizadeh et al. (2016) identified a naturalized accession of asparagus from Iran with 45S and 5S signals overlapping on the short arm of a submetacentric chromosome. The authors did not find a metacentric chromosome harboring a terminal 45S rDNA signal and explained this observation by a chromosome rearrangement.

In this work we detected 5S rDNA locus on submetacentric chromosome sorted from peak V, which was characterized with markers linked to sex gene, suggesting that the sex chromosome harbors 5S rDNA. Judging from its morphology, the chromosome may correspond to chromosome L4 of Deng et al. (2012). Löptien (1979) assigned sex locus to chromosome L5 by means of trisomics. However, Reamon-Büttner et al. (1999) did not find higher number of FISH signals with probes for 45S and 5S rDNA on L5 in a trisomic male, suggesting the absence of rDNA locus on the sex chromosome. Deng et al. (2012) described L5 as a metacentric chromosome that could be identified by FISH with a probe for 45S rDNA. We found 45S rDNA at the distal end of a metacentric chromosome, which was sorted from peak IV. This chromosome could be the same as the one reported by Deng et al. (2012).

Clearly, there is no general agreement concerning the number and chromosome location of rDNA loci in asparagus. However, according to our results, the sex locus could be located in a submetacentric chromosome which harbors 5S rDNA locus, and which can be sorted from peak V. The discrepancies could be due to intraspecific chromosome polymorphism, or difficulties in localizing rDNA probes on small chromosomes of asparagus. In fact, Mousavizadeh et al. (2016) noted that it was difficult to obtain good mitotic metaphase chromosome spreads and clear visualization of FISH signals in polyploid asparagus. As spatial resolution, sensitivity and specificity of FISH can be increased by using flow-sorted chromosomes as templates (Valárik et al., 2004; Doležel et al., 2011), the protocol for chromosome sorting developed in this study may facilitate long-range mapping of DNA sequences in asparagus to characterize asparagus genetic resources and support phylogenetic studies.

The genetic map reported in this work comprises 10 linkage groups, corresponding to the haploid chromosome number of asparagus. Based on the SSR markers mapped in each LG we were able to assign the LGs to individual peaks on flow karyotype. Importantly, genetic mapping confirmed that peak III comprised two different chromosomes and that the region delineated by peaks V and VI represents three chromosomes because markers assigned to these peaks mapped to three different LGs: LG5, LG10, and LG6. We have mapped the sex trait on LG5, which was assigned to peak V. This observation agrees with Löptien (1979), who identified L5 chromosome as the sex chromosome. Thus the genetic mapping together with flow karyotyping and chromosome sorting suggest that peak V represents the sex chromosome of asparagus.

Although sex is an important trait in asparagus production, codominant markers linked to sex are not yet available. Highly repetitive sequences were detected in the sex locus region using a BAC library, and cytogenetic analysis by FISH suggested that the locus was located near the centromere of chromosome L5 (Telgmann-Rauber et al., 2007). The authors recognized that map-based cloning in pericentromeric regions is difficult and suggested to use alternatives approaches for sex gene identification. An attractive strategy to reach this objective is to sequence the chromosome carrying sex locus. The recent progress in chromosome genomics makes it possible to obtain reference quality *de novo* assemblies from flow-sorted chromosomes (Thind et al., 2017) and the results obtained in this work provide an opportunity to develop such assembly from the asparagus sex chromosome.

In addition to sex determination, there is an urgent need to map other agronomically important traits of garden asparagus, such as early yield, spear color, spear size, spear quality, tight heads and disease resistance. However, the published attempts to map agronomic traits have been scarce. In our breeding program, we are evaluating the population from this study and several other mapping populations for different agronomic traits. These efforts will be supported by the ability to isolate asparagus chromosomes which will be used for targeted marker development from chromosomes carrying genes of agronomic importance as well as for gene isolation. The advantage of this approach has already been well documented in several crops, including wheat and barley (Sánchez-Martín et al., 2016; Thind et al., 2017).

## Data Statement

The raw sequencing data for individual samples has been submitted to the Sequence Read Archive (SRA) of the NCBI (SRP151603 BioProject PRJNA478227) https://www.ncbi.nlm.nih.gov/sra/SRP151603.

## Author Contributions

RM, JG, TM, and JD conceived and designed the study. RM and JG developed the plant material. VG performed the DNA extraction and contributed to the linkage map construction. PaC performed tGBS data analysis and constructed genetic linkage map. RM phenotyped the population and generated SSR data for map construction. JG and TM advised on linkage map construction. RM, JV, and PeC optimized protocol for flow cytometric chromosome analysis and sorting. RM performed the SSR amplification in flow-shorted chromosomes. MK and PeC advised in PCR analysis using flow-shorted chromosomes. MK performed the FISH experiments. RM, JG, PaC, and JD drafted the manuscript. TM critically read the manuscript. All authors read and approved the final version of the manuscript.

## Conflict of Interest Statement

The authors declare that the research was conducted in the absence of any commercial or financial relationships that could be construed as a potential conflict of interest.
