# Supplementary material for: Integration of Genetic and Cytogenetic Maps and Identification of Sex Chromosome in Garden Asparagus (Asparagus officinalis L.)
Source: Front Plant Sci. 2018 Jul 31;9:1068. doi: 10.3389/fpls.2018.01068 (PMC6079222; doi:10.3389/fpls.2018.01068)
Supplement: Supplementary file 1 [file Table_1.pdf]

Supplementary Material

Integration of Genetic and Cytogenetic Maps and Identification of Sex Chromosome in Garden Asparagus (*Asparagus officinalis* L.)

Roberto Moreno[1], Patricia Castro[1], Jan Vrána[2], Marie Kubaláková[2], Petr Cápal[2], Verónica García[1], Juan Gil[1], Teresa Millán[1*], Jaroslav Doležel[2*]

* **Correspondence:** Corresponding authors: dolezel@ueb.cas.cz; teresa.millan@uco.es

Supplementary Table 1. List of Simple Sequence Repeat (SSR) markers employed in this study. Markers in bold were included in the genetic map

SSR name	Forward Primer	Reverse Primer	Ta	Reference	Segregation*
**asp_c10809**	**GCTCCTCATGCCGTAAATGT**	**CGACCAAGTCCAAGAAAGGA**	**58ºC**	**Mercati et al. 2013**	**Heterozygous in PS010 (1:1)**
asp_c11315	AGATCTCCTCCTCCTCGTCC	GCCTCACCAAGGACCACTT	58ºC	Mercati et al. 2013	NP
asp_c11938	TTCTCGGGCTCGTCTTTCT	TCCATTACATCTACCGACGGA	58ºC	Mercati et al. 2013	NP
**asp_c11969**	**ACAGTGTGGAAGTTAGGCCG**	**CTCTTCCTCCTTCGAAACCC**	**58ºC**	**Mercati et al. 2013**	**Heterozygous in PS010 (1:1)**
**asp_c11979**	**CAGAAGGTGTATTGTTTGCCC**	**CCTCTTCATCTTGGCTTTCA**	**58ºC**	**Mercati et al. 2013**	**Heterozygous in PS010 (1:1)**
**asp_c12534**	**CCGCAGTGGATGAAGCTATT**	**TGCATCCACGTAACTAGCGA**	**58ºC**	**Mercati et al. 2013**	**Heterozygous in PS010 (1:1)**
asp_c12796	TGAATCGGGATCAAATTAGAGG	CGTTATCCTCACGACCCAAT	58ºC	Mercati et al. 2013	NP
asp_c12877	GAACGCATAGTACATGGCAAA	TTGATATCGATCTTGCTCGC	58ºC	Mercati et al. 2013	NP

asp_c1319	TGCTCAAGGCGTATGTGAAG	ACTGATTCTCGCTTTGCAGG	58°C	Mercati et al. 2013	NP
**asp_c13301**	**CATTACCTCCACGCTGTCCT**	**GACGATCCCTCTTCTGTGGA**	**58°C**	**Mercati et al. 2013**	**Heterozygous in WN124 (1:1)**
asp_c1367	GATGGAGGTTTGTACGGCCT	GTGGAGGTGAATCCGAGAAC	58°C	Mercati et al. 2013	NP
**asp_c1390**	**ACCTACGACATACGATGCCC**	**CTCACATGCGCTTGGAAAC**	**58°C**	**Mercati et al. 2013**	**Heterozygous in WN124 (1:1)**
**asp_c1401**	**AATGGTTGCCAATGGAGAAG**	**GCCTGCAGTGTCATCAGTGT**	**58°C**	**Mercati et al. 2013**	**Heterozygous in PS010 (1:1)**
**asp_c14231**	**CCACAGGATGCAAGTCCTTC**	**AGAGAGACTCGGGCTCATTG**	**58°C**	**Mercati et al. 2013**	**Heterozygous in WN124 (1:1)**
asp_c1505	ATCCCACGCACTGGTAAATC	AGGATATGGTTATGGCGGTG	58°C	Mercati et al. 2013	NP
asp_c15627	CTCTCATTGTTCGAAACGAGC	TGCTGCGATGCTAGAGAAGA	58°C	Mercati et al. 2013	NP
asp_c168	ATCATCGGCCACTGCTGAG	TGCAACAACCTACCAAGACG	58°C	Mercati et al. 2013	NP
asp_c16828	AGAAGGAAGAGAACCATGCG	ATGTTGGGTTGATGGGTTTG	58°C	Mercati et al. 2013	NP
asp_c17381	AGGGCTCCCAGTATCCAGTC	TTCATTGAACATGGCATTCG	58°C	Mercati et al. 2013	NP
**asp_c17476**	**AAGCCAGCCACAAGAACCTA**	**AAGAGCCTTGGCTAGCGTTT**	**58°C**	**Mercati et al. 2013**	**Heterozygous in PS010 (1:1)**
**asp_c17769**	**CTTCCCATCCATCTCATCTTTC**	**TCCGATAGTCACTCCCTCCA**	**58°C**	**Mercati et al. 2013**	**Heterozygous in PS010 (1:1)**
asp_c1779	CTGTGACATTAGCACAACTTAGCA	CAACCTTTCCTCGGAACGTA	58°C	Mercati et al. 2013	NP
asp_c17875	AGCTGAGCAAGTCCCAACTG	CTTCTACTGCTGCTTCCGCT	58°C	Mercati et al. 2013	NP
asp_c20351	CGAATAGATATTGATCCGCCT	GCCTTTACATCGTGAATGGTT	58°C	Mercati et al. 2013	NP
asp_c2065	ATGTCGACGATGATGCTCAG	GTACTGCGATATGCCGATGC	58°C	Mercati et al. 2013	NP
asp_c20893	AACCAGAGGTGTCTGCATTTG	CCCTCATCAGAAACAGCTTCA	58°C	Mercati et al. 2013	NP
asp_c2122	TCAGCCTCCTCTCTTGCTTC	AGTCAAAGAAGGACCCGGAG	58°C	Mercati et al. 2013	NP
asp_c21312	CCTCCAGTTCCCATCAGAAG	GGCTATAACCGTGGAGGAGG	58°C	Mercati et al. 2013	NP
asp_c22306	GATCATCATCTTGCGCATTG	AGAGGAAGCACGAGGAAGAA	58°C	Mercati et al. 2013	Heterozygous in PS010 (no fit segregation)

**asp_c22357**	**CAATCGACGGAGGAGAAAGA**	**AAGGCTTGTCTTCCATAGCG**	**58°C**	**Mercati et al. 2013**	**Heterozygous in both parents (1:1:1:1)**
asp_c2370	AGCCTGCCATAATCCTTTCC	TCCCTCTCCACATCTCTTCG	58°C	Mercati et al. 2013	NP
**asp_c2736**	**GCCTACGACATACAATGCCC**	**CTCACATGCGCTTGGAAAC**	**58°C**	**Mercati et al. 2013**	**Heterozygous in WN124 (1:1)**
asp_c2771	CCACCTTGACAAAGACCCAC	CCTTGATGGCCATTTCTCAC	58°C	Mercati et al. 2013	NP
asp_c2848	CCTTGTTCCAAGAGCTTCGT	CAGCGATGGAGAGAGGTACG	58°C	Mercati et al. 2013	NP
asp_c3091	GAACACTGAGACCAGGCAGC	GAACTCGGGCAACAATTCAG	58°C	Mercati et al. 2013	NP
asp_c3803	TAAACTGATGGTGAGGCTCG	TTGTAGGCGGCAGGCTATT	58°C	Mercati et al. 2013	NP
asp_c45	GGAGGAATGCCGACAAGG	TTTCTTCAATCGATCTCCTGG	58°C	Mercati et al. 2013	Heterozygous in WN124 (no fit segregation)
asp_c4593	TCCTCCTTCGACACCTTCAG	GACTCCGGAATCGAGAAGC	58°C	Mercati et al. 2013	NP
**asp_c4789**	**ACTTCCAAAGTCGCACACAC**	**TTTGTTGTTTGTTAATTTGCTGTT**	**58°C**	**Mercati et al. 2013**	**Heterozygous in WN124 (1:1)**
asp_c5202	AGCTTCAGCAGCAGCAGTC	CTGTGATCCCAAGTAGTTGCTG	58°C	Mercati et al. 2013	Heterozygous in WN124 (no fit segregation)
asp_c5587	TTTGTGGAGGGAGAGGGAG	CCACAAACAACTTTGCATCC	58°C	Mercati et al. 2013	NP
**asp_c6215**	**CCCAGCTCATAAAGAGGAAACA**	**GAGTTCGCAAACAGAGGAGG**	**58°C**	**Mercati et al. 2013**	**Heterozygous in WN124 (1:1)**
asp_c6290	CGGCGATAAATTGAAAGACC	ACTCAAGAAGCCGGAGGAAT	58°C	Mercati et al. 2013	NP
asp_c6470	AGAAAGTCACGGGCCTCC	TATCCTCCTCCTGATTCGCA	58°C	Mercati et al. 2013	NP
asp_c6790	GGAGAGAGGTCAGCATCTGG	GCCTTCACAACCTCCTCAAC	58°C	Mercati et al. 2013	NP
asp_c7389	TTACTCTCCTACGGGCATGA	ACCTGAACGGTCGCAATTAG	58°C	Mercati et al. 2013	NP
asp_c753	ATATTATGGTGGTGGCCGTG	TTCAATTGAGGGTGCAGATG	58°C	Mercati et al. 2013	NP
asp_c8280	CAATCTCTCCCACAAGCTCA	GATTGCTGGATTTGGTGAGG	58°C	Mercati et al. 2013	NP
asp_c8724	ACCTCCACATTCTATACGTTCCA	TGCTCCGTAAGGAGATTTCG	58°C	Mercati et al. 2013	NP
asp_c8860	CACCAACCATCAGCAATCAC	GCGTTGGTCCAGTCATACG	58°C	Mercati et al. 2013	NP

asp_c9020	GCAGCCAACCCTAGAAACAA	TATTATGAGCCTGTGGCTGG	58°C	Mercati et al. 2013	NP
**asp_c923**	**CACAGGTAAGGGATTGCAGC**	**CCCAAGCTACTCCAAAGCAG**	**58°C**	**Mercati et al. 2013**	**Heterozygous in both parents (1:1:1:1)**
asp_c9454	TTCTCTGGTTGCTAAATAGAAAGAAA	AGGAGAATTCGACCACCCTC	58°C	Mercati et al. 2013	NP
asp_c957	TGATGAACCACTCAATACATTCG	TGTGTCTTGTTGTTGTGGTGC	58°C	Mercati et al. 2013	Heterozygous in PS010 (no fit segregation)
**asp_c9810**	**AGGCAGAAGCTGAAGAGGC**	**TTCTTGCTCTCTGTTCCAGC**	**58°C**	**Mercati et al. 2013**	**Heterozygous in PS010 (1:1)**
**TC1**	**AGGTGGAGAACAAATGGCTG**	**CGAGCTCAATTGAAATCCATAA**	**55°C**	**Caruso et al. 2008**	**Heterozygous in PS010 (1:1)**
**AG2**	**CCTCCTCGGCAATTTAATCA**	**CAGCTGCATCACGTTCTTGT**	**55°C**	**Caruso et al. 2008**	**Heterozygous in both parents (1:1:1:1)**
AGA1	CCGGTGCTTTGATTACTGCT	GATCATCATCTTGCGCATTG	55°C	Caruso et al. 2008	NP
TC2	GGCAGGATTAGGGTTTCG	TCTCGCTCACCTTCTCATCC	55°C	Caruso et al. 2008	NP
AAT1	CTTTTGCTTCTGAACGCTCC	TTGAAGGAGCCGTAAACTGG	55°C	Caruso et al. 2008	Heterozygous in PS010 (no fit segregation)
**AG3**	**TCCACCCCACAAAAAGAAAG**	**AGAAGTTGACGCCGTTGTCT**	**55°C**	**Caruso et al. 2008**	**Heterozygous in WN124 (1:1)**
**TC3**	**CACCATTTCAAATCCCCACT**	**GAGGCTAGAGCTCCGCTCAT**	**55°C**	**Caruso et al. 2008**	**Heterozygous in PS010 (1:1)**
AG5	GATTAATAAAGCGCCGCTGA	ACATAAGCCCATACTTGCGG	55°C	Caruso et al. 2008	NP
AG6	TCATCTGAAATGGCATCAGC	CGAGGCCTAGTGTGTGTTGA	55°C	Caruso et al. 2008	NP
**AG7**	**TTTTGCTCCGATCATTTTCA**	**CCTCTTCGTCTTCATCAGCC**	**55°C**	**Caruso et al. 2008**	**Heterozygous in both parents (1:1:1:1)**
TC4	AGAGAGGAAGTTGTCGCTCG	TGGGAAAATGGAAGAACCAA	55°C	Caruso et al. 2008	NP
TC5	CCCGATCCAAACCCATCC	GAAAATTCGATCGGAACCCT	55°C	Caruso et al. 2008	NP
**AG8**	**GATTGGGACCAACACAAACA**	**AGCAATGACTTGATCCCCAG**	**55°C**	**Caruso et al. 2008**	**Heterozygous in WN124 (1:1)**
TC6	CATGCCCTAAAATCTCCAAGA	GCCAGAGGCTGAAATAAACTG	55°C	Caruso et al. 2008	NP
**TC7**	**CGCCCCGAATCAACTAATAA**	**TACTGCGGAGGTATGTGGGT**	**55°C**	**Caruso et al. 2008**	**Heterozygous in PS010 (1:1)**

TC8	GGCTAGCCGAAAGAATCTCC	TCTTCCTCCTCCTCCTCCTC	55°C	Caruso et al. 2008	NP
**AG10**	**CGCCCTTGTTCTTCTTCTTG**	**CAGTTGTCTGCCGTCTTCAA**	**55°C**	**Caruso et al. 2008**	**Heterozygous in WN124 (1:1)**
AG11	AGGGGTCCGGATTAATTCAC	GTCCTTGGCCATTAGAGCTG	55°C	Caruso et al. 2008	NP
**TC9**	**GTGATTCAAGGGGGAAAGGT**	**TACACCAAAACCAGAAGGGC**	**55°C**	**Caruso et al. 2008**	**Heterozygous in both parents (1:1:1:1)**
AG12	GACTAGCGCCATGAGAAAGG	TTTTAGGGCATTTTAAACGCAT	55°C	Caruso et al. 2008	Heterozygous in both parents (no fit segregation)
ssr13	CGACCAGAGAAGGAAGGAG	CAACCACGCTCATAAGAAC	55°C	Li et al. 2016	NP
**ssr15**	**ATGATCCCTGAAGTTGTTG**	**GTTCCTCTACCAGCCAAG**	**60°C**	**Li et al. 2016**	**Heterozygous in both parents (1:1:1:1)**
ssr20	CTCTACTCAACACTCTCC	TCTCTCCCGCTCTCTATC	55°C	Li et al. 2016	NP
ssr22	TAAGCAACTCACTCACTATG	TGATGTGTGAAGGAGGAGG	55°C	Li et al. 2016	Heterozygous in both parents (no fit segregation)
ssr23	GAGAACATAATCCAGAGAAC	GACACTCGCACAACCTT	55°C	Li et al. 2016	NP
ssr26	CTCTCCAACAGCCTTCTCC	CGCAAGATTAGTGGTGGAAG	55°C	Li et al. 2016	NP
ssr28	TGTTGGTTGTTGGTGTGAG	AGTGGTTGTTGTGGAGAG	55°C	Li et al. 2016	NP
ssr32	TACTGACTTCCTTGTGCTTG	TCCCTCACAGAACTTACG	55°C	Li et al. 2016	NP
ssr37	TATGTTCCTTGCTTCCATG	CGGTAGAAGTGATTGTGTAT	55°C	Li et al. 2016	NP
ssr40	GCATATTTCTACTACGCCTCC	CAAACTAACCCTCAATCACTCG	55°C	Li et al. 2016	Heterozygous in both parents (no fit segregation)
ssr41	CGCATGGGAAGAGAGCTAAAGT	CTGTGGAGTTGAAGGTGAAGAT	55°C	Li et al. 2016	Heterozygous in WN124 (no fit segregation)
ssr42	TTGATACCATCTTGCTGCT	ACCCTCCTCAACAATCGCAG	55°C	Li et al. 2016	NP
ssr43	CTTGATGGAGCTGGTCTTGT	TTCTCCACCCTCAATCTCAATAC	55°C	Li et al. 2016	NP
ssr53	AAGGAGACGAGGAGGATGTG	TGTGATGCAGAGACGTATTAG	55°C	Li et al. 2016	NP

ssr56	GCTGCTAAGGGATATAGTGCCA	TATGGTTGCAGAGGATAGGT	55°C	Li et al. 2016	Heterozygous in WN124 (no fit segregation)
ssr63	TTAAGTCAGGTGGTGCTCTC	CTGGATTAGTGGTTGATGATG	55°C	Li et al. 2016	Heterozygous in PS010 (no fit segregation)
ssr64	AGAGCAGAAAACACCGAGAG	TGTTGTTCGACCGCCGTT	55°C	Li et al. 2016	NP
ssr66	GTGGGATGTTCAATCTCTATGT	CTACCGTCTGAGAGCTATTCTT	55°C	Li et al. 2016	Heterozygous in both parents (no fit segregation)
**ssr69**	**GGCTAATTGTGTTGGGAATCG**	**CCAACTAATCTACTGACACACG**	**55°C**	**Li et al. 2016**	**Heterozygous in PS010 (1:1)**
ssr73	GTGTCATTACTGTTGAAGC	TCCGATTTCTTTATCTCCC	55°C	Li et al. 2016	Heterozygous in both parents (no fit segregation)
ssr77	GGCCTGCATGTTCTTTATATC	GCTCATTCTCATCCACTCAT	55°C	Li et al. 2016	NP
ssr83	GAGTTGAGGCGAGGGACAT	GTTACTTTCGAGGAGGCCA	55°C	Li et al. 2016	NP

* NP, markers homozygous in both parents and not polymorphic in the population
