# Supplementary material for: Integration of Genetic and Cytogenetic Maps and Identification of Sex Chromosome in Garden Asparagus (Asparagus officinalis L.)
Source: Front Plant Sci. 2018 Jul 31;9:1068. doi: 10.3389/fpls.2018.01068 (PMC6079222; doi:10.3389/fpls.2018.01068)
Supplement: Supplementary file 2 [file Image_1.pdf]

Supplementary Material

Integration of Genetic and Cytogenetic Maps and Identification of Sex Chromosome in Garden Asparagus (*Asparagus officinalis* L.)

Roberto Moreno[1], Patricia Castro[1], Jan Vrána[2], Marie Kubaláková[2], Petr Cápal[2], Verónica García[1], Juan Gil[1], Teresa Millán[1*], Jaroslav Doležel[2*]

* **Correspondence:** Corresponding Authors: dolezel@ueb.cas.cz; teresa.millan@uco.es

Supplementary Figure 1. Genetic linkage map constructed in the asparagus population PS010 x WN124. Underlined and bold markers are markers linking parental maps. Bold markers are SSR markers.

.

PS010 LG1 **WN124 LG1**

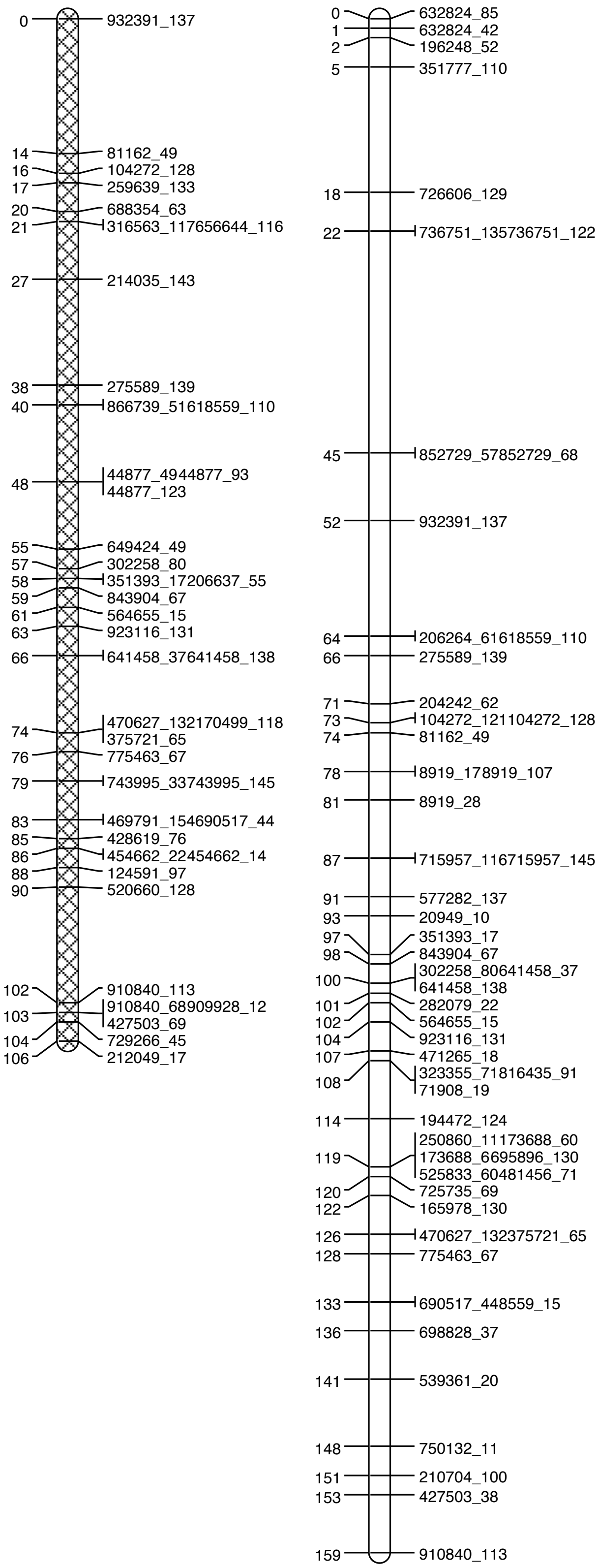

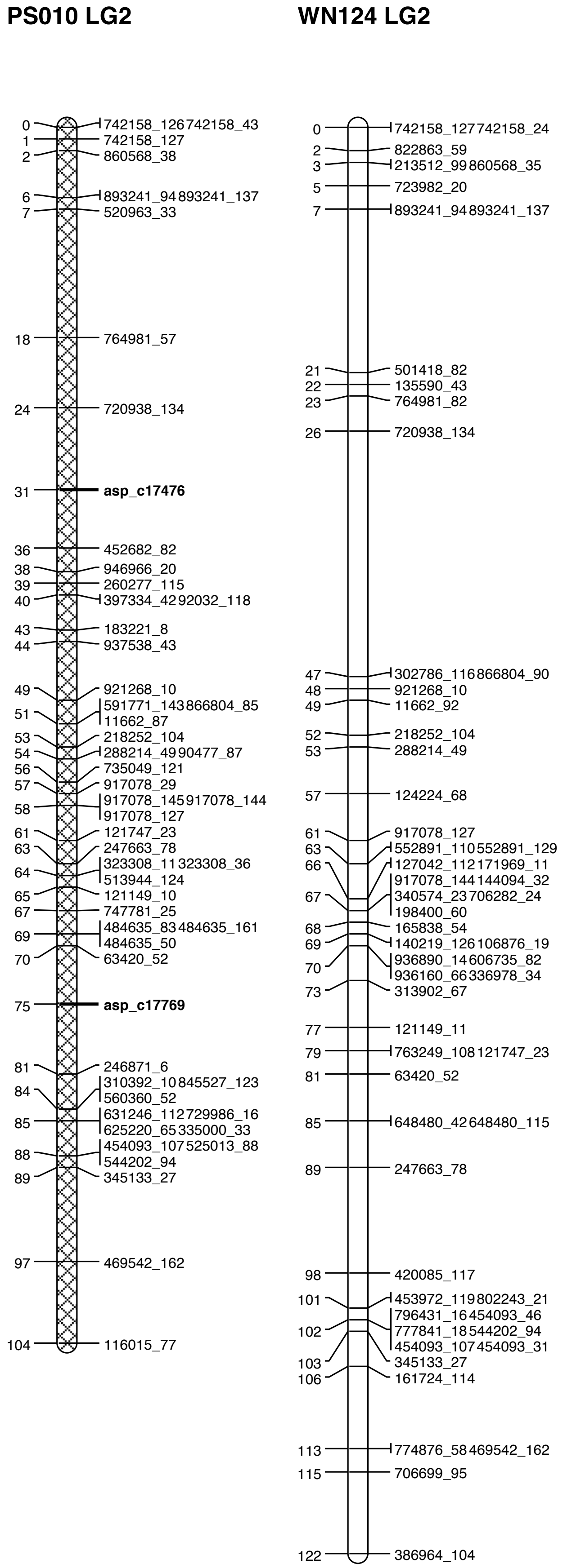
PS010 LG2
WN124 LG2
0 742158_126 742158_43
1 742158_127
2 860568_38
6 893241_94 893241_137
7 520963_33
18 764981_57
24 720938_134
31 asp_c17476
36 452682_82
38 946966_20
39 260277_115
40 397334_42 92032_118
43 183221_8
44 937538_43
49 921268_10
51 591771_143 866804_85
11662_87
53 218252_104
54 288214_49 90477_87
56 735049_121
57 917078_29
58 917078_145 917078_144
917078_127
61 121747_23
63 247663_78
64 323308_11 323308_36
513944_124
65 121149_10
67 747781_25
69 484635_83 484635_161
484635_50
70 63420_52
75 asp_c17769
81 246871_6
84 310392_10 845527_123
560360_52
85 631246_112 729986_16
625220_65 335000_33
88 454093_107 525013_88
544202_94
89 345133_27
97 469542_162
104 116015_77
0 742158_127 742158_24
2 822863_59
3 213512_99 860568_35
5 723982_20
7 893241_94 893241_137
21 501418_82
22 135590_43
23 764981_82
26 720938_134
47 302786_116 866804_90
48 921268_10
49 11662_92
52 218252_104
53 288214_49
57 124224_68
61 917078_127
63 552891_110 552891_129
66 127042_112 171969_11
67 917078_144 144094_32
340574_23 706282_24
198400_60
68 165838_54
69 140219_126 106876_19
70 936890_14 606735_82
936160_66 336978_34
73 313902_67
77 121149_11
79 763249_108 121747_23
81 63420_52
85 648480_42 648480_115
89 247663_78
98 420085_117
101 453972_119 802243_21
102 796431_16 454093_46
777841_18 544202_94
454093_107 454093_31
103 345133_27
106 161724_114
113 774876_58 469542_162
115 706699_95
122 386964_104

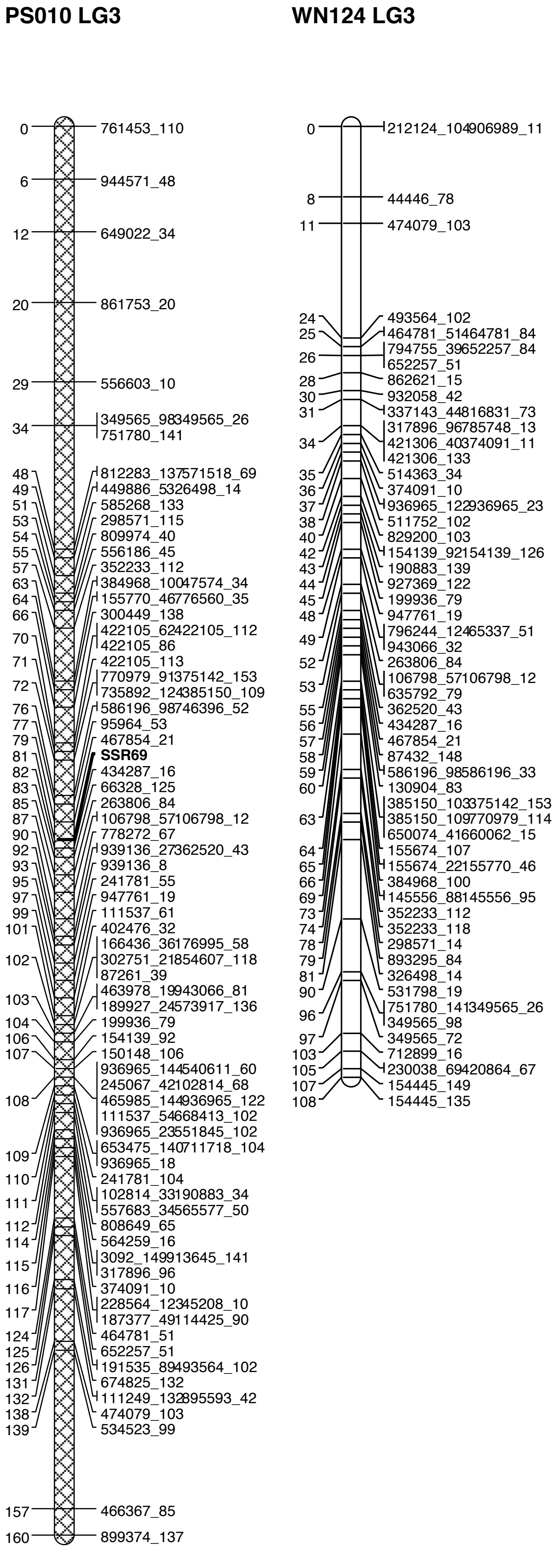
PS010 LG3
WN124 LG3
0 761453_110
6 944571_48
12 649022_34
20 861753_20
29 556603_10
34 349565_98349565_26
751780_141
48 812283_137571518_69
49 449886_5326498_14
51 585268_133
53 298571_115
54 809974_40
55 556186_45
57 352233_112
63 384968_10047574_34
64 155770_46776560_35
66 300449_138
70 422105_62422105_112
422105_86
71 422105_113
72 770979_91375142_153
735892_124385150_109
76 586196_98746396_52
77 95964_53
79 467854_21
81 SSR69
82 434287_16
83 66328_125
85 263806_84
87 106798_57106798_12
90 778272_67
92 939136_27362520_43
93 939136_8
95 241781_55
97 947761_19
99 111537_61
101 402476_32
102 166436_36176995_58
302751_21854607_118
87261_39
103 463978_19943066_81
189927_24573917_136
104 199936_79
106 154139_92
107 150148_106
108 936965_144540611_60
245067_42102814_68
465985_144936965_122
111537_54668413_102
936965_23551845_102
109 653475_140711718_104
936965_18
110 241781_104
111 102814_33190883_34
557683_34565577_50
112 808649_65
114 564259_16
115 3092_149913645_141
317896_96
116 374091_10
117 228564_12345208_10
187377_49114425_90
124 464781_51
125 652257_51
126 191535_89493564_102
131 674825_132
132 111249_132895593_42
138 474079_103
139 534523_99
157 466367_85
160 899374_137
0 212124_104906989_11
8 44446_78
11 474079_103
24 493564_102
25 464781_51464781_84
26 794755_39652257_84
652257_51
28 862621_15
30 932058_42
31 337143_44816831_73
34 317896_96785748_13
421306_40374091_11
421306_133
35 514363_34
36 374091_10
37 936965_122936965_23
38 511752_102
40 829200_103
42 154139_92154139_126
43 190883_139
44 927369_122
45 199936_79
48 947761_19
49 796244_12465337_51
943066_32
52 263806_84
53 106798_57106798_12
635792_79
55 362520_43
56 434287_16
57 467854_21
58 87432_148
59 586196_98586196_33
60 130904_83
63 385150_103375142_153
385150_109770979_114
650074_41660062_15
64 155674_107
65 155674_22155770_46
66 384968_100
69 145556_88145556_95
73 352233_112
74 352233_118
78 298571_14
79 893295_84
81 326498_14
90 531798_19
96 751780_141349565_26
349565_98
97 349565_72
103 712899_16
105 230038_69420864_67
107 154445_149
108 154445_135

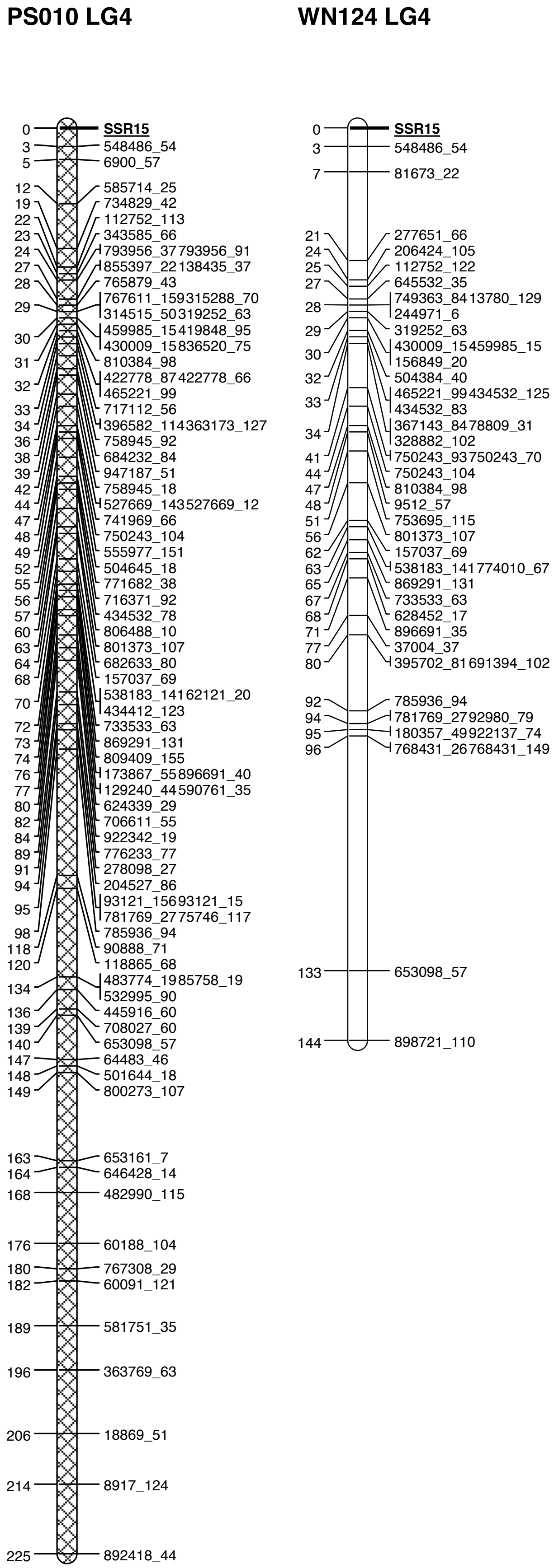
PS010 LG4
0 SSR15
3 548486_54
5 6900_57
12 585714_25
19 734829_42
22 112752_113
23 343585_66
24 793956_37793956_91
27 855397_22138435_37
28 765879_43
29 767611_159315288_70
314515_50319252_63
30 459985_15419848_95
430009_15836520_75
31 810384_98
32 422778_87422778_66
465221_99
33 717112_56
34 396582_114363173_127
36 758945_92
38 684232_84
39 947187_51
42 758945_18
44 527669_143527669_12
47 741969_66
48 750243_104
49 555977_151
52 504645_18
55 771682_38
56 716371_92
57 434532_78
60 806488_10
63 801373_107
64 682633_80
68 157037_69
70 538183_14162121_20
434412_123
72 733533_63
73 869291_131
74 809409_155
76 173867_55896691_40
77 129240_44590761_35
80 624339_29
82 706611_55
84 922342_19
89 776233_77
91 278098_27
94 204527_86
95 93121_15693121_15
781769_2775746_117
98 785936_94
118 90888_71
120 118865_68
134 483774_1985758_19
532995_90
136 445916_60
139 708027_60
140 653098_57
147 64483_46
148 501644_18
149 800273_107
163 653161_7
164 646428_14
168 482990_115
176 60188_104
180 767308_29
182 60091_121
189 581751_35
196 363769_63
206 18869_51
214 8917_124
225 892418_44
WN124 LG4
0 SSR15
3 548486_54
7 81673_22
21 277651_66
24 206424_105
25 112752_122
27 645532_35
28 749363_8413780_129
244971_6
29 319252_63
30 430009_15459985_15
156849_20
32 504384_40
33 465221_99434532_125
434532_83
34 367143_8478809_31
328882_102
41 750243_93750243_70
44 750243_104
47 810384_98
48 9512_57
51 753695_115
56 801373_107
62 157037_69
63 538183_141774010_67
65 869291_131
67 733533_63
68 628452_17
71 896691_35
77 37004_37
80 395702_81691394_102
92 785936_94
94 781769_2792980_79
95 180357_49922137_74
96 768431_26768431_149
133 653098_57
144 898721_110

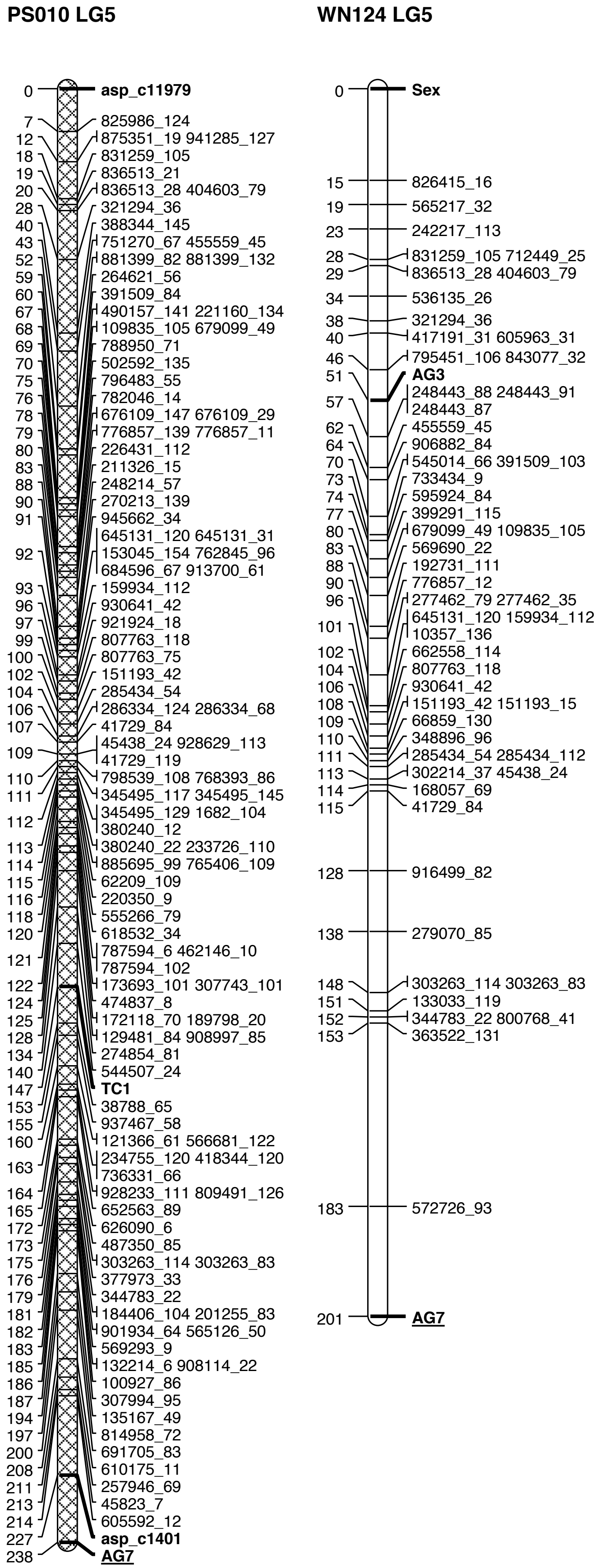
PS010 LG5
WN124 LG5
0 asp_c11979
7 825986_124
12 875351_19 941285_127
18 831259_105
19 836513_21
20 836513_28 404603_79
28 321294_36
40 388344_145
43 751270_67 455559_45
52 881399_82 881399_132
59 264621_56
60 391509_84
67 490157_141 221160_134
68 109835_105 679099_49
69 788950_71
70 502592_135
75 796483_55
76 782046_14
78 676109_147 676109_29
79 776857_139 776857_11
80 226431_112
83 211326_15
88 248214_57
90 270213_139
91 945662_34
92 645131_120 645131_31
153045_154 762845_96
684596_67 913700_61
93 159934_112
96 930641_42
97 921924_18
99 807763_118
100 807763_75
102 151193_42
104 285434_54
106 286334_124 286334_68
107 41729_84
109 45438_24 928629_113
41729_119
110 798539_108 768393_86
111 345495_117 345495_145
112 345495_129 1682_104
380240_12
113 380240_22 233726_110
114 885695_99 765406_109
115 62209_109
116 220350_9
118 555266_79
120 618532_34
121 787594_6 462146_10
787594_102
122 173693_101 307743_101
124 474837_8
125 172118_70 189798_20
128 129481_84 908997_85
134 274854_81
140 544507_24
147 TC1
153 38788_65
155 937467_58
160 121366_61 566681_122
163 234755_120 418344_120
736331_66
164 928233_111 809491_126
165 652563_89
172 626090_6
173 487350_85
175 303263_114 303263_83
176 377973_33
179 344783_22
181 184406_104 201255_83
182 901934_64 565126_50
183 569293_9
185 132214_6 908114_22
186 100927_86
187 307994_95
194 135167_49
197 814958_72
200 691705_83
208 610175_11
211 257946_69
213 45823_7
214 605592_12
227 asp_c1401
238 AG7
0 Sex
15 826415_16
19 565217_32
23 242217_113
28 831259_105 712449_25
29 836513_28 404603_79
34 536135_26
38 321294_36
40 417191_31 605963_31
46 795451_106 843077_32
51 AG3
57 248443_88 248443_91
248443_87
62 455559_45
64 906882_84
70 545014_66 391509_103
73 733434_9
74 595924_84
77 399291_115
80 679099_49 109835_105
83 569690_22
88 192731_111
90 776857_12
96 277462_79 277462_35
101 645131_120 159934_112
10357_136
102 662558_114
104 807763_118
106 930641_42
108 151193_42 151193_15
109 66859_130
110 348896_96
111 285434_54 285434_112
113 302214_37 45438_24
114 168057_69
115 41729_84
128 916499_82
138 279070_85
148 303263_114 303263_83
151 133033_119
152 344783_22 800768_41
153 363522_131
183 572726_93
201 AG7

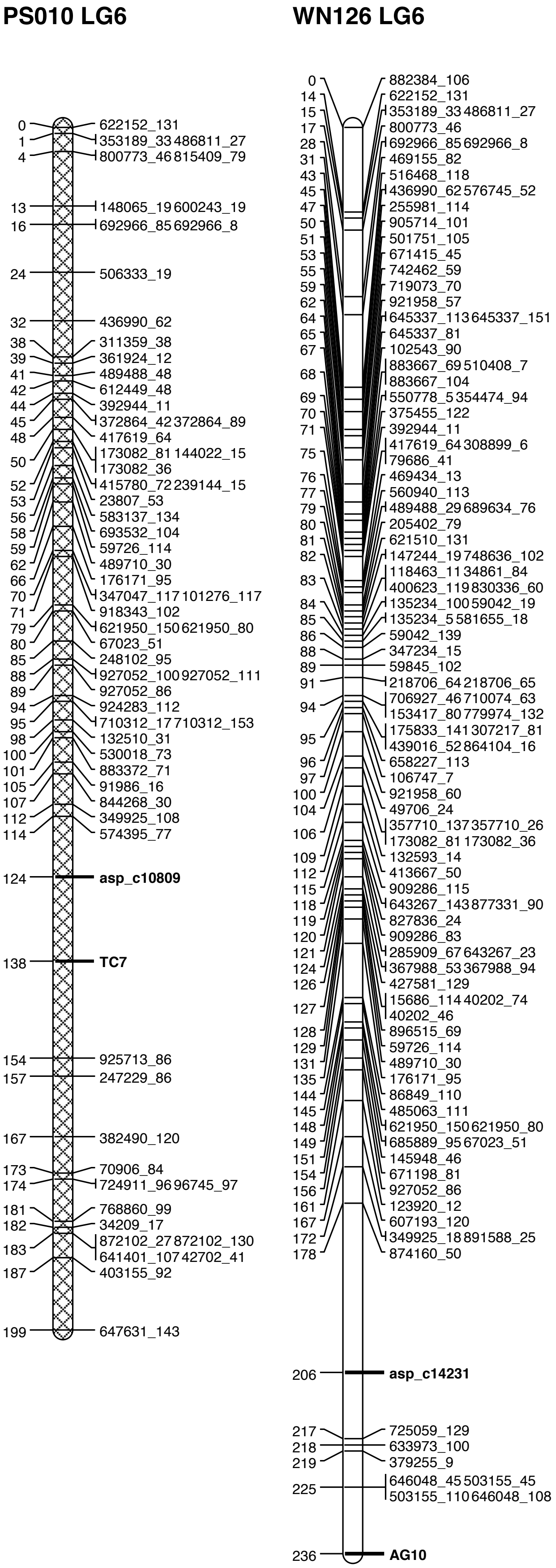

PS010 LG6
0 622152_131
1 353189_33 486811_27
4 800773_46 815409_79
13 148065_19 600243_19
16 692966_85 692966_8
24 506333_19
32 436990_62
38 311359_38
39 361924_12
41 489488_48
42 612449_48
44 392944_11
45 372864_42 372864_89
48 417619_64
50 173082_81 144022_15
173082_36
52 415780_72 239144_15
53 23807_53
56 583137_134
58 693532_104
59 59726_114
62 489710_30
66 176171_95
70 347047_117 101276_117
71 918343_102
79 621950_150 621950_80
80 67023_51
85 248102_95
88 927052_100 927052_111
89 927052_86
94 924283_112
95 710312_17 710312_153
98 132510_31
100 530018_73
101 883372_71
105 91986_16
107 844268_30
112 349925_108
114 574395_77
124 asp_c10809
138 TC7
154 925713_86
157 247229_86
167 382490_120
173 70906_84
174 724911_96 96745_97
181 768860_99
182 34209_17
183 872102_27 872102_130
641401_107 42702_41
187 403155_92
199 647631_143
WN126 LG6
0 882384_106
14 622152_131
15 353189_33 486811_27
17 800773_46
28 692966_85 692966_8
31 469155_82
43 516468_118
45 436990_62 576745_52
47 255981_114
50 905714_101
51 501751_105
53 671415_45
55 742462_59
59 719073_70
62 921958_57
64 645337_113 645337_151
65 645337_81
67 102543_90
68 883667_69 510408_7
883667_104
69 550778_5 354474_94
70 375455_122
71 392944_11
75 417619_64 308899_6
79686_41
76 469434_13
77 560940_113
79 489488_29 689634_76
80 205402_79
81 621510_131
82 147244_19 748636_102
83 118463_11 34861_84
400623_119 830336_60
84 135234_100 59042_19
85 135234_5 581655_18
86 59042_139
88 347234_15
89 59845_102
91 218706_64 218706_65
94 706927_46 710074_63
153417_80 779974_132
95 175833_141 307217_81
439016_52 864104_16
96 658227_113
97 106747_7
100 921958_60
104 49706_24
106 357710_137 357710_26
173082_81 173082_36
109 132593_14
112 413667_50
115 909286_115
118 643267_143 877331_90
119 827836_24
120 909286_83
121 285909_67 643267_23
124 367988_53 367988_94
126 427581_129
127 15686_114 40202_74
40202_46
128 896515_69
129 59726_114
131 489710_30
135 176171_95
144 86849_110
145 485063_111
148 621950_150 621950_80
149 685889_95 67023_51
151 145948_46
154 671198_81
156 927052_86
161 123920_12
167 607193_120
172 349925_18 891588_25
178 874160_50
206 asp_c14231
217 725059_129
218 633973_100
219 379255_9
225 646048_45 503155_45
503155_110 646048_108
236 AG10

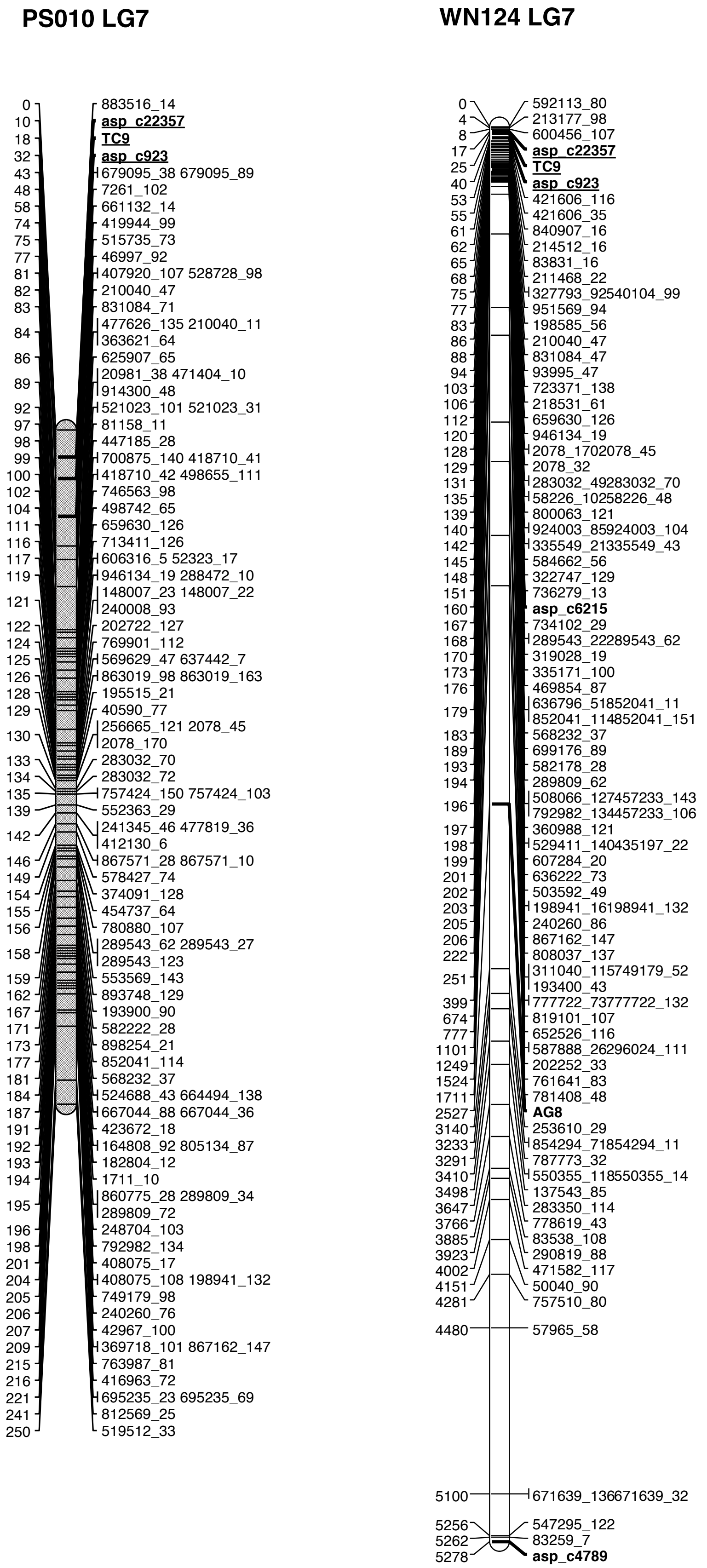

PS010 LG7
0 883516_14
10 asp_c22357
18 TC9
32 asp_c923
43 679095_38 679095_89
48 7261_102
58 661132_14
74 419944_99
75 515735_73
77 46997_92
81 407920_107 528728_98
82 210040_47
83 831084_71
84 477626_135 210040_11 363621_64
86 625907_65
89 20981_38 471404_10 914300_48
92 521023_101 521023_31
97 81158_11
98 447185_28
99 700875_140 418710_41
100 418710_42 498655_111
102 746563_98
104 498742_65
111 659630_126
116 713411_126
117 606316_5 52323_17
119 946134_19 288472_10
121 148007_23 148007_22 240008_93
122 202722_127
124 769901_112
125 569629_47 637442_7
126 863019_98 863019_163
128 195515_21
129 40590_77
130 256665_121 2078_45 2078_170
133 283032_70
134 283032_72
135 757424_150 757424_103
139 552363_29
142 241345_46 477819_36 412130_6
146 867571_28 867571_10
149 578427_74
154 374091_128
155 454737_64
156 780880_107
158 289543_62 289543_27 289543_123
159 553569_143
162 893748_129
167 193900_90
171 582222_28
173 898254_21
177 852041_114
181 568232_37
184 524688_43 664494_138
187 667044_88 667044_36
191 423672_18
192 164808_92 805134_87
193 182804_12
194 1711_10
195 860775_28 289809_34 289809_72
196 248704_103
198 792982_134
201 408075_17
204 408075_108 198941_132
205 749179_98
206 240260_76
207 42967_100
209 369718_101 867162_147
215 763987_81
216 416963_72
221 695235_23 695235_69
241 812569_25
250 519512_33
WN124 LG7
0 592113_80
4 213177_98
8 600456_107
17 asp_c22357
25 TC9
40 asp_c923
53 421606_116
55 421606_35
61 840907_16
62 214512_16
65 83831_16
68 211468_22
75 327793_92540104_99
77 951569_94
83 198585_56
86 210040_47
88 831084_47
94 93995_47
103 723371_138
106 218531_61
112 659630_126
120 946134_19
128 2078_1702078_45
129 2078_32
131 283032_49283032_70
135 58226_10258226_48
139 800063_121
140 924003_85924003_104
142 335549_21335549_43
145 584662_56
148 322747_129
151 736279_13
160 asp_c6215
167 734102_29
168 289543_22289543_62
170 319028_19
173 335171_100
176 469854_87
179 636796_51852041_11 852041_114852041_151
183 568232_37
189 699176_89
193 582178_28
194 289809_62
196 508066_127457233_143 792982_134457233_106
197 360988_121
198 529411_140435197_22
199 607284_20
201 636222_73
202 503592_49
203 198941_16198941_132
205 240260_86
206 867162_147
222 808037_137
251 311040_115749179_52 193400_43
399 777722_73777722_132
674 819101_107
777 652526_116
1101 587888_26296024_111
1249 202252_33
1524 761641_83
1711 781408_48
2527 AG8
3140 253610_29
3233 854294_71854294_11
3291 787773_32
3410 550355_118550355_14
3498 137543_85
3647 283350_114
3766 778619_43
3885 83538_108
3923 290819_88
4002 471582_117
4151 50040_90
4281 757510_80
4480 57965_58
5100 671639_136671639_32
5256 547295_122
5262 83259_7
5278 asp_c4789

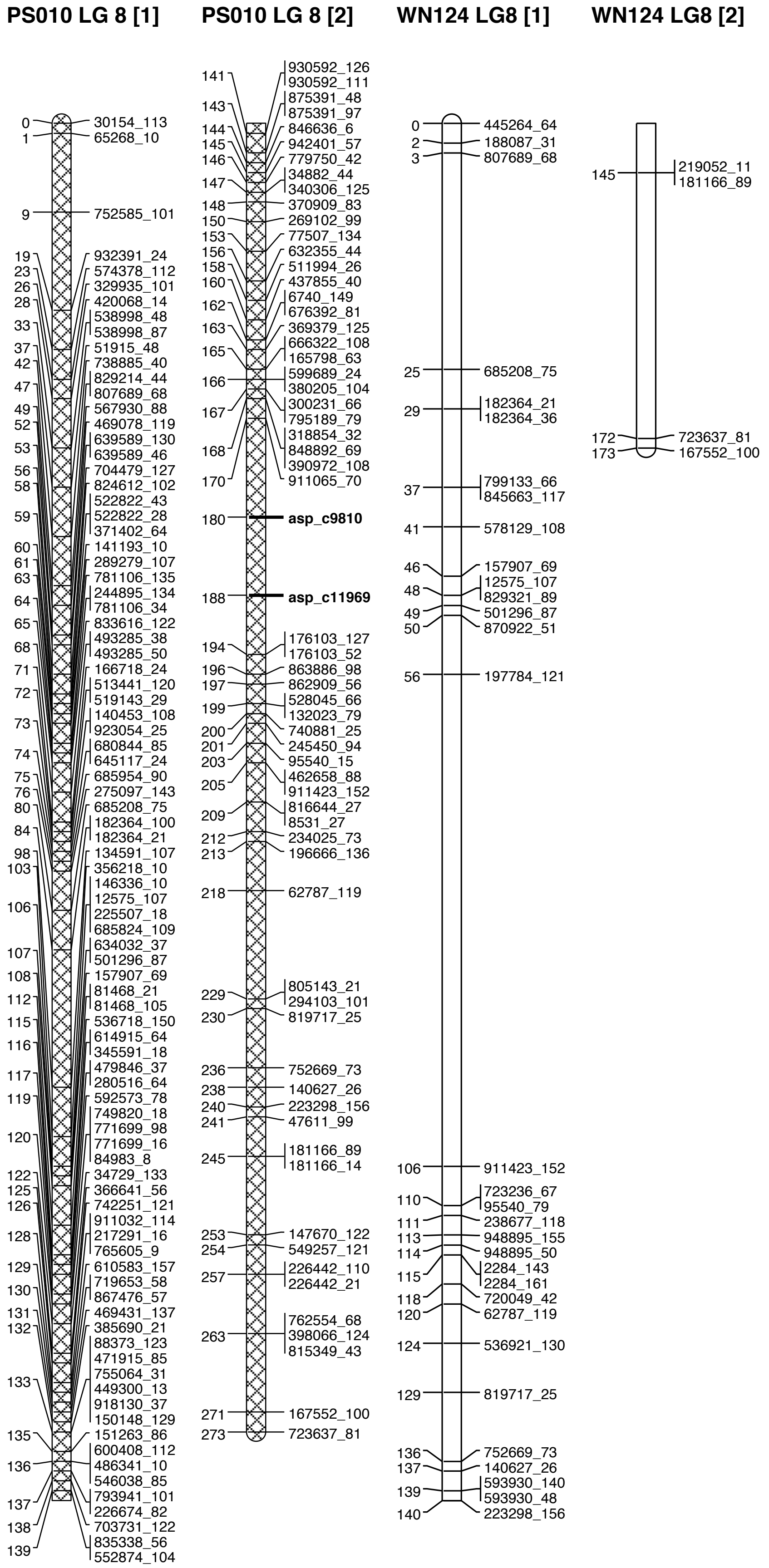

PS010 LG 8 [1]
0 30154_113
1 65268_10
9 752585_101
19 23 26 28 33 37 42 47 49 52 53 56 58 59 60 61 63 64 65 68 71 72 73 74 75 76 80 84 98 103 106 107 108 112 115 116 117 119 120 122 125 126 128 129 130 131 132 133 135 136 137 138 139
932391_24
574378_112
329935_101
420068_14
538998_48
538998_87
51915_48
738885_40
829214_44
807689_68
567930_88
469078_119
639589_130
639589_46
704479_127
824612_102
522822_43
522822_28
371402_64
141193_10
289279_107
781106_135
244895_134
781106_34
833616_122
493285_38
493285_50
166718_24
513441_120
519143_29
140453_108
923054_25
680844_85
645117_24
685954_90
275097_143
685208_75
182364_100
182364_21
134591_107
356218_10
146336_10
12575_107
225507_18
685824_109
634032_37
501296_87
157907_69
81468_21
81468_105
536718_150
614915_64
345591_18
479846_37
280516_64
592573_78
749820_18
771699_98
771699_16
84983_8
34729_133
366641_56
742251_121
911032_114
217291_16
765605_9
610583_157
719653_58
867476_57
469431_137
385690_21
88373_123
471915_85
755064_31
449300_13
918130_37
150148_129
151263_86
600408_112
486341_10
546038_85
793941_101
226674_82
703731_122
835338_56
552874_104
PS010 LG 8 [2]
141 143 144 145 146 147 148 150 153 156 158 160 162 163 165 166 167 168 170
930592_126
930592_111
875391_48
875391_97
846636_6
942401_57
779750_42
34882_44
340306_125
370909_83
269102_99
77507_134
632355_44
511994_26
437855_40
6740_149
676392_81
369379_125
666322_108
165798_63
599689_24
380205_104
300231_66
795189_79
318854_32
848892_69
390972_108
911065_70
180 asp_c9810
188 asp_c11969
194 196 197 199 200 201 203 205 209 212 213
176103_127
176103_52
863886_98
862909_56
528045_66
132023_79
740881_25
245450_94
95540_15
462658_88
911423_152
816644_27
8531_27
234025_73
196666_136
218 62787_119
229 230
805143_21
294103_101
819717_25
236 752669_73
238 140627_26
240 223298_156
241 47611_99
245 181166_89
181166_14
253 147670_122
254 549257_121
257 226442_110
226442_21
263 762554_68
398066_124
815349_43
271 167552_100
273 723637_81
WN124 LG8 [1]
0 445264_64
2 188087_31
3 807689_68
25 685208_75
29 182364_21
182364_36
37 799133_66
845663_117
41 578129_108
46 157907_69
48 12575_107
829321_89
49 501296_87
50 870922_51
56 197784_121
106 911423_152
110 723236_67
95540_79
111 238677_118
113 948895_155
114 948895_50
115 2284_143
2284_161
118 720049_42
120 62787_119
124 536921_130
129 819717_25
136 752669_73
137 140627_26
139 593930_140
593930_48
140 223298_156
WN124 LG8 [2]
145 219052_11
181166_89
172 723637_81
173 167552_100

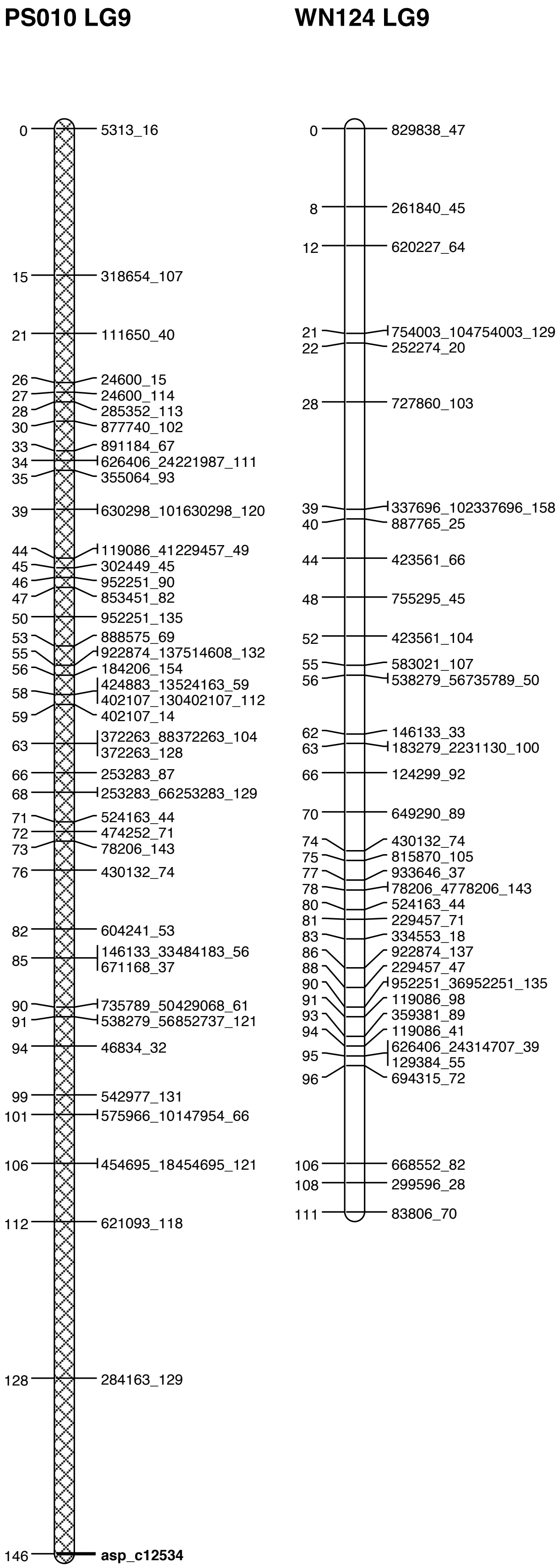
PS010 LG9
0 5313_16
15 318654_107
21 111650_40
26 24600_15
27 24600_114
28 285352_113
30 877740_102
33 891184_67
34 626406_24221987_111
35 355064_93
39 630298_101630298_120
44 119086_41229457_49
45 302449_45
46 952251_90
47 853451_82
50 952251_135
53 888575_69
55 922874_137514608_132
56 184206_154
58 424883_13524163_59
402107_130402107_112
59 402107_14
63 372263_88372263_104
372263_128
66 253283_87
68 253283_66253283_129
71 524163_44
72 474252_71
73 78206_143
76 430132_74
82 604241_53
85 146133_33484183_56
671168_37
90 735789_50429068_61
91 538279_56852737_121
94 46834_32
99 542977_131
101 575966_10147954_66
106 454695_18454695_121
112 621093_118
128 284163_129
146 asp_c12534
WN124 LG9
0 829838_47
8 261840_45
12 620227_64
21 754003_104754003_129
22 252274_20
28 727860_103
39 337696_102337696_158
40 887765_25
44 423561_66
48 755295_45
52 423561_104
55 583021_107
56 538279_56735789_50
62 146133_33
63 183279_2231130_100
66 124299_92
70 649290_89
74 430132_74
75 815870_105
77 933646_37
78 78206_4778206_143
80 524163_44
81 229457_71
83 334553_18
86 922874_137
88 229457_47
90 952251_36952251_135
91 119086_98
93 359381_89
94 119086_41
95 626406_24314707_39
129384_55
96 694315_72
106 668552_82
108 299596_28
111 83806_70

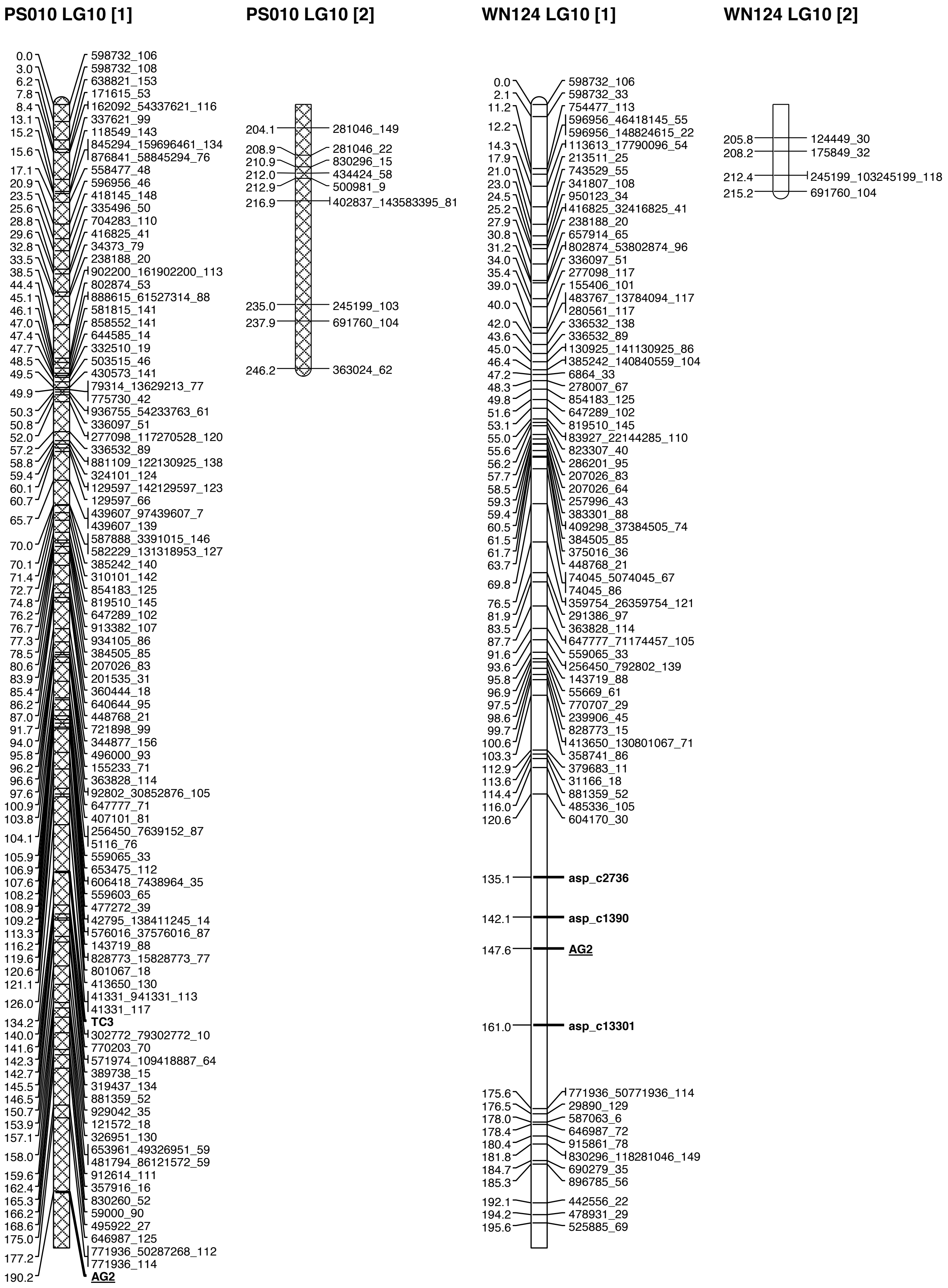

PS010 LG10 [1]
0.0 598732_106
3.0 598732_108
6.2 638821_153
7.8 171615_53
8.4 162092_54337621_116
13.1 337621_99
15.2 118549_143
15.6 845294_159696461_134
876841_58845294_76
17.1 558477_48
20.9 596956_46
23.5 418145_148
25.6 335496_50
28.8 704283_110
29.6 416825_41
32.8 34373_79
33.5 238188_20
38.5 902200_161902200_113
44.4 802874_53
45.1 888615_61527314_88
46.1 581815_141
47.0 858552_141
47.4 644585_14
47.7 332510_19
48.5 503515_46
49.5 430573_141
49.9 79314_13629213_77
775730_42
50.3 936755_54233763_61
50.8 336097_51
52.0 277098_117270528_120
57.2 336532_89
58.8 881109_122130925_138
59.4 324101_124
60.1 129597_142129597_123
60.7 129597_66
65.7 439607_97439607_7
439607_139
70.0 587888_3391015_146
582229_131318953_127
70.1 385242_140
71.4 310101_142
72.7 854183_125
74.8 819510_145
76.2 647289_102
76.7 913382_107
77.3 934105_86
78.5 384505_85
80.6 207026_83
83.9 201535_31
85.4 360444_18
86.2 640644_95
87.0 448768_21
91.7 721898_99
94.0 344877_156
95.8 496000_93
96.2 155233_71
96.6 363828_114
97.6 92802_30852876_105
100.9 647777_71
103.8 407101_81
104.1 256450_7639152_87
5116_76
105.9 559065_33
106.9 653475_112
107.6 606418_7438964_35
108.2 559603_65
108.9 477272_39
109.2 42795_138411245_14
113.3 576016_37576016_87
116.2 143719_88
119.6 828773_15828773_77
120.6 801067_18
121.1 413650_130
126.0 41331_941331_113
41331_117
134.2 TC3
140.0 302772_79302772_10
141.6 770203_70
142.3 571974_109418887_64
142.7 389738_15
145.5 319437_134
146.5 881359_52
150.7 929042_35
153.9 121572_18
157.1 326951_130
158.0 653961_49326951_59
481794_86121572_59
159.6 912614_111
162.4 357916_16
165.3 830260_52
166.2 59000_90
168.6 495922_27
175.0 646987_125
177.2 771936_50287268_112
771936_114
190.2 AG2
PS010 LG10 [2]
204.1 281046_149
208.9 281046_22
210.9 830296_15
212.0 434424_58
212.9 500981_9
216.9 402837_143583395_81
235.0 245199_103
237.9 691760_104
246.2 363024_62
WN124 LG10 [1]
0.0 598732_106
2.1 598732_33
11.2 754477_113
12.2 596956_46418145_55
596956_148824615_22
14.3 113613_17790096_54
17.9 213511_25
21.0 743529_55
23.0 341807_108
24.5 950123_34
25.2 416825_32416825_41
27.9 238188_20
30.8 657914_65
31.2 802874_53802874_96
34.0 336097_51
35.4 277098_117
39.0 155406_101
40.0 483767_13784094_117
280561_117
42.0 336532_138
43.6 336532_89
45.0 130925_141130925_86
46.4 385242_140840559_104
47.2 6864_33
48.3 278007_67
49.8 854183_125
51.6 647289_102
53.1 819510_145
55.0 83927_22144285_110
55.6 823307_40
56.2 286201_95
57.7 207026_83
58.5 207026_64
59.3 257996_43
59.4 383301_88
60.5 409298_37384505_74
61.5 384505_85
61.7 375016_36
63.7 448768_21
69.8 74045_5074045_67
74045_86
76.5 359754_26359754_121
81.9 291386_97
83.5 363828_114
87.7 647777_71174457_105
91.6 559065_33
93.6 256450_792802_139
95.8 143719_88
96.9 55669_61
97.5 770707_29
98.6 239906_45
99.7 828773_15
100.6 413650_130801067_71
103.3 358741_86
112.9 379683_11
113.6 31166_18
114.4 881359_52
116.0 485336_105
120.6 604170_30
135.1 asp_c2736
142.1 asp_c1390
147.6 AG2
161.0 asp_c13301
175.6 771936_50771936_114
176.5 29890_129
178.0 587063_6
178.4 646987_72
180.4 915861_78
181.8 830296_118281046_149
184.7 690279_35
185.3 896785_56
192.1 442556_22
194.2 478931_29
195.6 525885_69
WN124 LG10 [2]
205.8 124449_30
208.2 175849_32
212.4 245199_103245199_118
215.2 691760_104
